# Personalizing Peri-Intubation Oxygenation in Patients at Risk of Acute Hypoxemic Respiratory Failure: A Phenotype-Driven Narrative Review of High-Flow Nasal Oxygen and Non-Invasive Ventilation

**DOI:** 10.3390/jpm16090455

**Published:** 2026-08-29

**Authors:** Daniele Salvatore Paternò, Luigi La Via, Rossella Moltisanti, Antonio Putaggio, Angela Maria Piccolo, Giorgia Maria Noce, Roberta Scuto, Gilberto Duarte-Medrano, Natalia Nuño-Lámbarri, Emilia Concetta Lo Giudice, Massimiliano Sorbello

**Affiliations:** 1 Department of Anesthesia and Intensive Care, “Giovanni Paolo II” Hospital, 97100 Ragusa, Italy; danielesalvatore.paterno@asp.rg.it (D.S.P.); rossella.moltisanti@asp.rg.it (R.M.); antonio.putaggio@asp.rg.it (A.P.); angelamaria.piccolo@asp.rg.it (A.M.P.); massimiliano.sorbello@unikore.it (M.S.); 2 Department of General Surgery and Medical-Surgical Specialties, University of Catania, 95123 Catania, Italy; 3 School of Anesthesia and Intensive Care, “Kore” University, 94100 Enna, Italy; giorgiamaria.noce@unikorestudent.it (G.M.N.); roberta.scuto@unikorestudent.it (R.S.); emilia.logiudice@unikore.it (E.C.L.G.); 4 Department of Anesthesiology, Medica Sur Clinic & Foundation, Mexico City 11510, Mexico; gduartem@medicasur.org.mx; 5 Department of Surgery, Faculty of Medicine, The National Autonomous University of Mexico (UNAM), Mexico City 11510, Mexico; nnunol@medicasur.org.mx

**Keywords:** high-flow nasal oxygen, non-invasive ventilation, pre-oxygenation, apneic oxygenation, tracheal intubation, acute hypoxemic respiratory failure, airway management, personalized medicine

## Abstract

Tracheal intubation in patients at risk of acute hypoxemic respiratory failure (AHRF) carries a high risk of life-threatening desaturation, and the choice of peri-intubation oxygenation strategy critically influences patient safety. This narrative review synthesizes current evidence on non-invasive oxygenation techniques—high-flow nasal oxygen (HFNO), non-invasive ventilation (NIV), and their combination—across the pre-oxygenation, apneic, and awake-intubation phases of airway management. We examine the physiological mechanisms underlying each modality, appraise landmark randomized trials and meta-analyses (including PREOXI, OPTINIV, and OPTIMASK), and address disease-specific considerations in chronic obstructive pulmonary disease, heart failure, interstitial lung disease, severe obesity, obstructive sleep apnea, and obstetric, pediatric, and trauma populations. The evidence supports a phenotype-driven hierarchy rather than a single dominant technique: NIV—optionally combined with HFNO for apneic oxygenation—is preferred in severely hypoxemic critically ill patients, whereas HFNO alone is adequate for many moderately hypoxemic or non-hypoxemic patients. Progressive hypercapnia limits apneic oxygenation, particularly in chronic CO_2_ retainers, underscoring the value of continuous CO_2_ monitoring. Persistent under-implementation of NIV-based pre-oxygenation reveals a gap between evidence and practice. Individualized, physiology-guided oxygenation—aligned with the goals of personalized peri-procedural medicine—offers the greatest potential to reduce peri-intubation morbidity.

## 1. Introduction

Tracheal intubation is among the most common life-saving procedures performed in critically ill patients, yet it remains a high-risk intervention. The international INTUBE study, which prospectively documented intubation practice across 29 countries, reported peri-intubation cardiovascular instability in over 40% of critically ill patients, severe hypoxemia in approximately 9%, and cardiac arrest in 3.1% [1]. These figures are even higher in patients presenting with—or at risk of—acute hypoxemic respiratory failure (AHRF), in whom reduced functional residual capacity, increased oxygen consumption, ventilation–perfusion mismatch, and limited physiological reserve compress the safety margin available to the operator. In this population, even a brief desaturation episode during airway instrumentation may precipitate cardiac arrest, hypoxic brain injury, or death [2,3].

The clinical phenotype of the patient at risk of peri-intubation AHRF is heterogeneous but recognizable. It includes critically ill patients requiring emergency intubation for respiratory or cardiovascular deterioration; patients with chronic cardio-respiratory disease such as chronic obstructive pulmonary disease (COPD), heart failure, or interstitial lung disease (ILD), whose baseline reserve is already eroded; patients with severe obesity or obstructive sleep apnea (OSA), in whom anatomical and physiological factors converge to accelerate desaturation; high-risk obstetric patients, particularly in the context of emergency cesarean section or pre-eclampsia complicated by pulmonary edema; and trauma patients, in whom hypovolemia, thoracic injury, or traumatic brain injury further compromise oxygen delivery [3,4,5]. Across these populations, the shared challenge is to maintain adequate arterial oxygenation continuously—from the pre-oxygenation phase, through induction and apnea, until successful airway securement.

Conventional facemask pre-oxygenation, while standard, has well-recognized limitations in patients at risk of AHRF. The mask must be removed at the moment of laryngoscopy, interrupting oxygen delivery during the very interval in which the patient is most vulnerable to desaturation. Mask leaks and patient discomfort further reduce the achievable end-tidal oxygen fraction, particularly in obese or distressed patients. These shortcomings have driven a substantial evolution in peri-intubation oxygenation practice over the past decade, centered on two non-invasive modalities: high-flow nasal oxygen (HFNO) and non-invasive ventilation (NIV/NIPPV) [6,7,8].

HFNO delivers heated, fully humidified oxygen at flow rates up to 70 L·min^−1^ via wide-bore nasal cannulae, generating low-level positive airway pressure, washing out anatomical dead space, and—crucially—remaining in situ throughout laryngoscopy. NIV provides higher airway pressures and active pressure support, advantages that become decisive in severely hypoxemic patients with significant atelectasis or ventilation–perfusion mismatch. More recently, the combination of NIV for pre-oxygenation with HFNO maintained throughout the apneic interval, formalized in the OPTINIV trial [9], has emerged as a strategy that exploits the complementary strengths of both modalities. The relative positioning of these three approaches—and the choice of one over another in specific clinical phenotypes—is the subject of an active and clinically consequential debate, recently informed by large randomized trials and network meta-analyses [10,11].

The aim of this narrative review is to synthesize the available evidence on peri-intubation oxygenation strategies in patients at risk of AHRF, with explicit focus on HFNO, NIV, and the combined approach. We organize the discussion by phase of airway management—pre-oxygenation, apneic oxygenation, and awake tracheal intubation—and by clinical population, emphasizing disease-specific physiological considerations relevant to chronic cardio-respiratory disease, obesity/OSA, high-risk obstetrics, pediatrics, and trauma. Throughout, the underlying principle is that modality selection should be guided by patient phenotype and severity of hypoxemia rather than by institutional habit. Framed within the paradigm of personalized (precision) medicine, this approach treats peri-intubation oxygenation not as a one-size-fits-all protocol but as a decision tailored to the individual patient’s respiratory phenotype, comorbidities, and physiological reserve—matching the right modality (HFNO, NIV, or their combination) to the right patient at the right moment.

## 2. Methods

### 2.1. Review Design

This work is presented as a structured narrative review. The choice of a narrative—rather than systematic—synthesis reflects the nature of the available evidence on peri-intubation oxygenation in patients at risk of acute hypoxemic respiratory failure (AHRF): a heterogeneous body of literature spanning diverse clinical settings (operating room, intensive care unit, emergency department, pre-hospital), patient populations, intervention protocols, comparators, and outcome definitions. A narrative synthesis allows for clinically meaningful interpretation across these dimensions, which a strictly quantitative or systematic approach could not accommodate within a single manuscript. While this review does not meet the formal requirements of a systematic review (e.g., PRISMA-compliant protocol registration, duplicate independent screening, or formal risk-of-bias assessment), the search and selection process was structured and is explicitly reported below to support transparency and reproducibility.

### 2.2. Literature Search

A literature search was conducted in PubMed (MEDLINE), Embase, and the Cochrane Library from inception through May 2026. Search terms combined controlled vocabulary (MeSH/EMTREE) and free-text terms covering the intervention domain—“high-flow nasal oxygen”, “high-flow nasal cannula”, “HFNO”, “HFNC”, “THRIVE”, “transnasal humidified rapid-insufflation ventilatory exchange”, “non-invasive ventilation”, “NIV”, “NIPPV”, “CPAP”, “BiPAP”—combined with the procedural domain—“pre-oxygenation”, “preoxygenation”, “apneic oxygenation”, “apnoeic oxygenation”, “rapid sequence induction”, “rapid sequence intubation”, “awake tracheal intubation”, “peri-intubation”—and with population terms relevant to AHRF risk: “acute hypoxemic respiratory failure”, “critically ill”, “chronic obstructive pulmonary disease”, “heart failure”, “interstitial lung disease”, “obesity”, “obstructive sleep apnea”, “pregnancy”, “obstetric”, “pediatric”, “trauma”, and “emergency”. Reference lists of retrieved articles, relevant systematic reviews and network meta-analyses, and authoritative clinical practice guidelines (ESA/ESICM, ASA, DAS, OAA, SIAARTI) were hand-searched for additional studies of relevance.

### 2.3. Eligibility Criteria

Eligible publications included randomized controlled trials, prospective and retrospective observational studies, systematic reviews and meta-analyses, narrative reviews, clinical practice guidelines, and physiological or mechanistic studies addressing HFNO and/or NIV in the peri-intubation phases—pre-oxygenation, apneic oxygenation, or awake tracheal intubation—of patients at risk of AHRF. Both adult and pediatric studies were considered. Studies in elective surgical populations were retained when the underlying physiology (reduced functional reserve, accelerated desaturation, anatomically or physiologically difficult airway) was directly transferable to the AHRF-risk setting; studies focused on post-extubation respiratory support, procedural sedation outside the airway management context, or perioperative oxygenation unrelated to intubation were excluded. Case reports and conference abstracts were generally excluded, except for reports describing rare but clinically relevant adverse events. Only English-language publications were considered.

### 2.4. Synthesis Approach

Selected studies were synthesized narratively and organized along two axes: (i) phase of airway management—pre-oxygenation, apneic oxygenation, and awake tracheal intubation; and (ii) clinical population, with explicit attention to chronic cardio-respiratory disease, severe obesity and OSA, high-risk obstetrics, pediatrics, critically ill patients, and trauma. For each topic, the evidence was summarized with attention to study design, comparator, key outcomes, and limitations. Where applicable, contrasting findings between studies were explicitly discussed. No formal quantitative synthesis (meta-analysis) was performed. The strength of evidence supporting clinical considerations was qualitatively appraised based on study design, methodological rigor, and consistency across studies, drawing on the conceptual framework of the Oxford Centre for Evidence-Based Medicine (OCEBM) Levels of Evidence (2011), without applying formal quantitative grading. To make the strength of the underlying evidence explicit, clinical considerations throughout this review are qualified as resting on (i) direct randomised evidence obtained in the peri-intubation setting, (ii) indirect or extrapolated clinical evidence—randomised data from adjacent settings, non-randomised peri-intubation studies, or subgroup analyses—or (iii) physiological rationale and expert interpretation in the absence of comparative clinical data. This descriptor is applied alongside, and informed by, the OCEBM Levels of Evidence framework [12]. Where a recommendation rests on category (ii) or (iii), this is stated explicitly in the text.

A summary of the principal studies informing the present synthesis is provided in Table 1, organized by phase of airway management, with key information on author, year, design, population, comparator, main outcomes, and OCEBM level of evidence.

## 3. Non-Invasive Peri-Intubation Oxygenation: Mechanisms and Physiology

### 3.1. High-Flow Nasal Oxygen: Clinical Setup and Technical Features

HFNO is a non-invasive oxygenation modality originally developed for hypoxemic patients in the intensive care unit and subsequently extended to a broad range of clinical settings, including the operating room, the emergency department, and the pre-hospital environment [6,7]. Its applicability across the peri-intubation continuum—from pre-oxygenation, through apneic oxygenation during airway instrumentation, to support during awake tracheal intubation—has been the focus of intensive investigation over the past decade [36,37].

**Figure 1:**
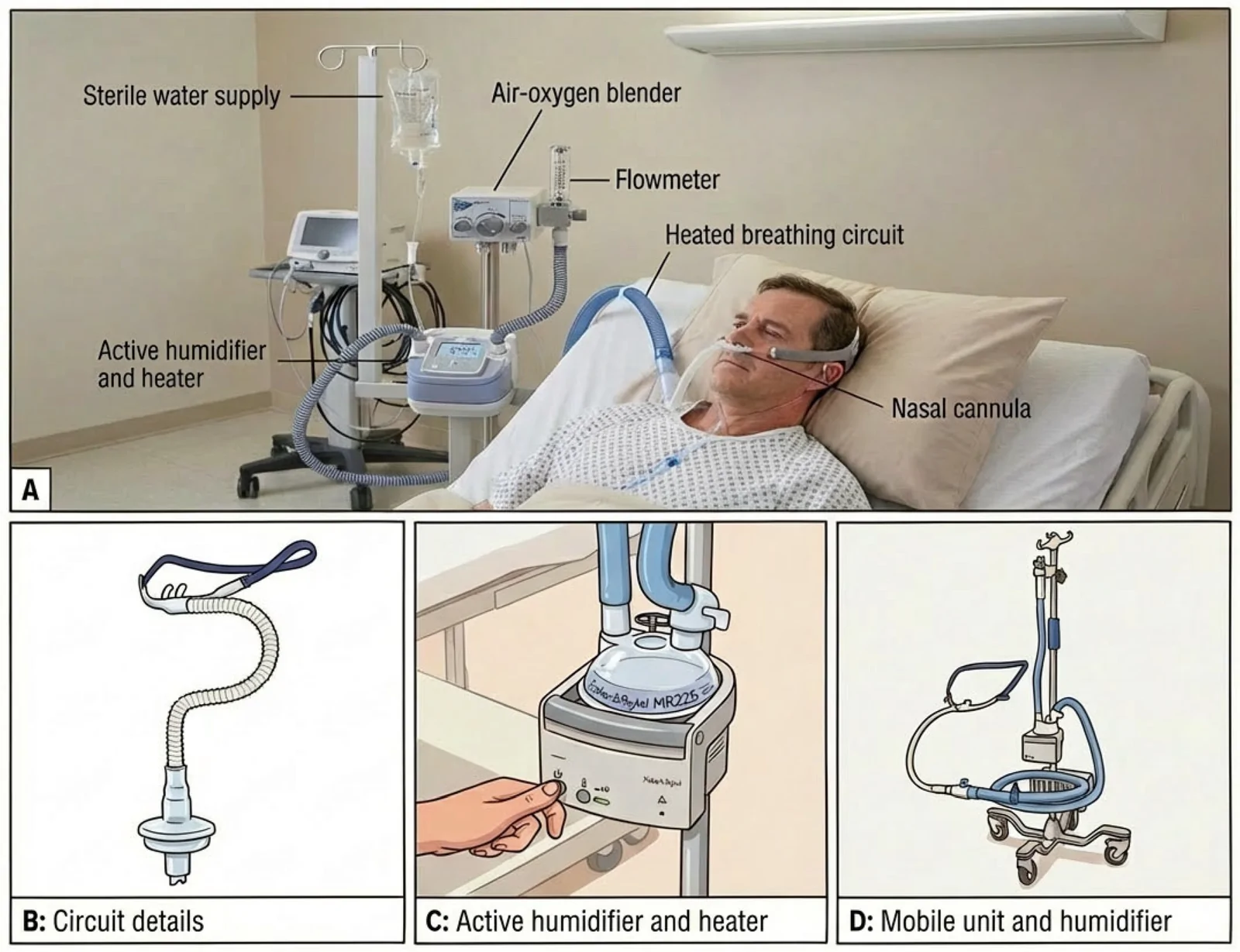
Components of the high-flow nasal oxygen (HFNO) system. (**A**) Complete clinical setup at the bedside, showing the air–oxygen blender, flowmeter, active humidifier and heater, sterile water supply, heated breathing circuit and wide-bore nasal cannula; (**B**) circuit details; (**C**) active humidifier and heater; (**D**) mobile unit and humidifier.

The HFNO system delivers heated and fully humidified oxygen—maintained at 34–37 °C with 100% relative humidity (equivalent to 44 mg H_2_O·L^−1^)—through dedicated wide-bore nasal cannulae at flow rates ranging from 20 to 70 L·min^−1^, with an adjustable FiO_2_ from 0.21 to 1.0 [7,30,38]. The system comprises a soft silicone nasal interface with openings wider than those of conventional nasal cannulae, a flow meter, an air–oxygen blender, a single heated inspiratory circuit with minimal condensation, an active humidifier, and an oxygen inlet (Figure 1).

### 3.2. HFNO: Mechanism of Action and Physiological Benefits

HFNO exerts its physiological effects through four interrelated mechanisms (Table 2). First, at flow rates exceeding 60 L·min^−1^, HFNO reduces anatomical dead space and promotes washout of expired carbon dioxide from the upper airways, thereby minimizing CO_2_ rebreathing [30,31]. Second, because the delivered flow rate exceeds the patient’s peak inspiratory flow, dilution of oxygen with ambient air is prevented, ensuring close correspondence between set and delivered FiO_2_. Third, although HFNO does not form a closed circuit, the high flow rate limits expiratory air outflow, generating low-level positive airway pressure of approximately 2.7–7.4 cmH_2_O—analogous to (but substantially weaker than) PEEP—which promotes alveolar recruitment, attenuates atelectasis, and reduces ventilation–perfusion mismatch [31,32,33,39]. Fourth, the delivery of adequately heated and humidified gas prevents mucosal dryness, reduces the energy expenditure associated with airway gas conditioning, protects the mucociliary epithelium, and improves patient tolerability compared with both conventional facemask oxygen and NIV [38,40,41].

**Figure 2:**
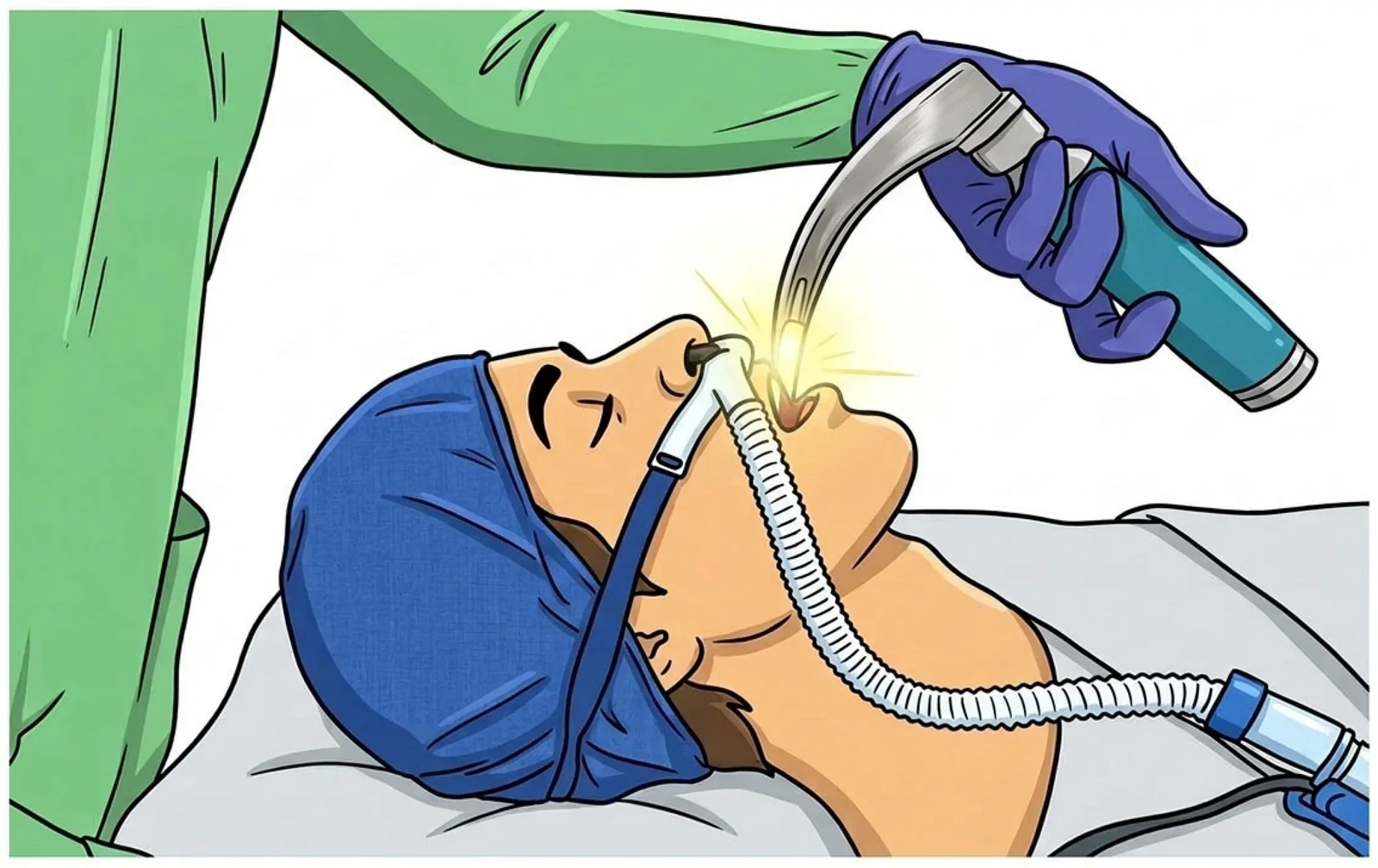
Maintenance of high-flow nasal oxygen during peri-intubation airway management. The wide-bore nasal cannula remains in situ, delivering continuous heated, humidified high-flow oxygen and providing apneic oxygenation while the operator performs laryngoscopy and tracheal intubation.

Collectively, these mechanisms translate into a more favorable respiratory pattern, with reduced respiratory rate, decreased work of breathing, and increased tidal volume [6,30]. From the peri-intubation perspective, the most clinically consequential property of HFNO is that the nasal cannulae remain in situ throughout laryngoscopy and tube passage, enabling uninterrupted apneic oxygenation during the very interval in which conventional facemask or NIV-based strategies must be interrupted (Figure 2).

The clinical benefits of HFNO vary according to the interface used. Natalini and colleagues demonstrated that, in tracheostomized patients, a minimum flow rate of 50 L·min^−1^ is required to achieve adequate oxygenation and meaningful reduction in work of breathing, whereas patients receiving HFNO via nasal cannulae achieve comparable effects at flow rates of 30 L·min^−1^ [34]. This difference reflects the fact that, with tracheal delivery, the reduction in anatomical dead space and inspiratory resistance is attenuated, necessitating higher flows to achieve equivalent benefit—a consideration relevant to peri-intubation management of tracheostomized patients requiring tube replacement.

### 3.3. Non-Invasive Ventilation: Physiological Principles Relevant to Peri-Intubation Oxygenation

NIV—encompassing continuous positive airway pressure (CPAP) and bilevel positive airway pressure (BiPAP/NIPPV)—delivers positive airway pressure through a sealed interface (typically a full-facemask or a helmet). Unlike HFNO, NIV provides active pressure support in addition to a constant end-expiratory pressure, and the magnitude of positive pressure achievable is substantially greater (typically 5–15 cmH_2_O of PEEP, plus 5–15 cmH_2_O of inspiratory pressure support) [42,43,44]. These features yield three physiological advantages of direct relevance to peri-intubation oxygenation in patients at risk of AHRF: (i) more effective alveolar recruitment with a reduction in intrapulmonary shunt and improvement in ventilation–perfusion matching, particularly in patients with significant atelectasis or pulmonary edema; (ii) active augmentation of tidal volume during the pre-oxygenation phase, which accelerates denitrogenation and increases the end-tidal oxygen fraction achieved before induction; and (iii) unloading of inspiratory muscles, reducing oxygen consumption in patients with high work of breathing [42,43,44].

The principal limitation of NIV in the peri-intubation context is interface-related: the sealed mask must be removed at the moment of laryngoscopy, interrupting both oxygen delivery and positive airway pressure precisely when the patient is most vulnerable to desaturation and derecruitment. Helmet interfaces partially mitigate this issue but introduce other constraints (rebreathing risk, less consistent leak control, patient claustrophobia) [44]. Patient tolerability of NIV is generally inferior to that of HFNO, particularly during prolonged application and in awake, anxious, or distressed patients [40,43].

The complementarity between HFNO and NIV—the former offering uninterrupted apneic oxygenation but limited positive pressure, the latter offering robust pressure support but requiring removal at laryngoscopy—provides the physiological rationale for the combined approach (NIV for pre-oxygenation, HFNO maintained throughout the apneic interval), formalized in the OPTINIV trial and discussed in Section 4.4 [9].

### 3.4. Contraindications and Cautions

HFNO is contraindicated in cases of severe nasal obstruction, profuse epistaxis, recent facial trauma, recent nasal surgery, significantly raised intracranial pressure, and skull base fractures, owing to the risk of pneumocephalus [45,46,47]. For NIV, it is clinically more useful to frame contraindications around the bedside decision than as a list of diagnoses. Even when severe hypoxemia would otherwise favour positive-pressure pre-oxygenation, NIV should be withheld or abandoned in the presence of active vomiting or substantial upper gastrointestinal bleeding; a major aspiration risk; inability to protect the airway or depressed consciousness; severe agitation or non-cooperation; facial trauma, burns, or anatomy precluding an adequate mask seal; undrained pneumothorax; and recent upper airway, oesophageal, or gastric surgery [42,43,44]. Haemodynamic considerations are addressed separately in Section 3.5, since they modify rather than simply prohibit the use of positive pressure. In each of these situations HFNO is frequently the only practicable non-invasive option, and the clinical question becomes how to shorten the apneic interval and prepare for rapid airway securement rather than which pre-oxygenation device is theoretically superior.

### 3.5. Haemodynamic Consequences of Peri-Intubation Respiratory Support

A phenotype-driven strategy that considers only gas exchange is incomplete. Positive airway pressure improves oxygenation by recruiting collapsed alveoli and reducing the work of breathing, but it also raises intrathoracic pressure, which reduces venous return and right ventricular preload and may increase right ventricular afterload. In a patient with adequate intravascular volume these effects are typically inconsequential and may even be beneficial, by reducing left ventricular afterload in acute cardiogenic pulmonary oedema. In the hypovolaemic or vasoplegic patient the same physiology can precipitate abrupt hypotension, and this occurs at the moment when induction agents and the transition to positive-pressure ventilation are already compromising haemodynamics. Peri-intubation cardiovascular collapse is at least as frequent as severe desaturation in critically ill patients and carries comparable prognostic weight [2,3].

Three phenotypes deserve particular caution. In septic or haemorrhagic shock, the reduction in venous return produced by NIV may be poorly tolerated, and volume resuscitation and vasopressor readiness should precede rather than follow the decision to apply positive pressure. In right ventricular dysfunction and in pulmonary hypertension, increases in transpulmonary pressure may further impair right ventricular ejection; here the lower and less controlled airway pressure generated by HFNO—approximately 2.7–7.4 cmH_2_O with the mouth closed [32,39]—may be haemodynamically advantageous even though it is less effective at recruitment. Conversely, in acute cardiogenic pulmonary oedema the afterload-reducing effect of positive pressure is desirable and argues in favour of NIV. The practical implication is that the respiratory phenotype determines which modality best prevents desaturation, while the haemodynamic phenotype determines how safely that modality can be delivered; the two must be assessed together, and where they conflict, the strategy should be chosen to protect the more threatened system. It should be acknowledged that no randomised trial has compared pre-oxygenation modalities using peri-intubation haemodynamic collapse as a primary endpoint; the considerations in this section therefore rest on physiological rationale and observational data rather than on direct randomised evidence.

### 3.6. Terminology

Throughout this review we use the term HFNO consistently to refer to high-flow nasal oxygen therapy, emphasizing the oxygenation modality rather than the interface alone (the literature also uses “HFNC”, high-flow nasal cannula, interchangeably). Where THRIVE (transnasal humidified rapid-insufflation ventilatory exchange) is referred to specifically, this denotes the application of HFNO during apnea for the purpose of extending safe apnea time, as originally described by Patel and Nouraei [20]. We use NIV as the umbrella term encompassing both CPAP and BiPAP/NIPPV; where the distinction is clinically relevant, the specific modality is named.

## 4. Pre-Oxygenation

### 4.1. General Principles for Patients at Risk of AHRF

Adequate pre-oxygenation is the single most effective maneuver available to the operator to extend safe apnea time and to mitigate peri-intubation desaturation [48,49]. The physiological objective is to replace nitrogen with oxygen in the functional residual capacity (FRC), thereby maximizing the alveolar oxygen reservoir available during apnea. In healthy adults, three to five minutes of tidal-volume breathing of 100% oxygen through a well-fitted facemask achieves adequate denitrogenation and permits an apneic interval of seven to ten minutes without significant desaturation [48,50]. An end-tidal oxygen fraction (EtO_2_ ≥ 90%) is widely accepted as the operational target confirming effective pre-oxygenation [48,49].

In patients at risk of AHRF, these reassuring figures do not apply. Reduced FRC (obesity, pregnancy, atelectasis, ILD), increased oxygen consumption (sepsis, hyperdynamic circulation, pregnancy), ventilation–perfusion mismatch and intrapulmonary shunt (pneumonia, ARDS, pulmonary edema), and impaired diffusion (ILD, fibrosis) each compress the safety margin. In severely hypoxemic critically ill patients, the time to desaturation below 90% may be reduced to less than 60 s [3,51]. Consequently, the choice of pre-oxygenation modality is not a matter of habit but a clinically consequential decision that should be guided by the patient’s phenotype and the severity of baseline hypoxemia.

### 4.2. HFNO vs. Conventional Facemask

Intratracheal FiO_2_ during HFNO increases proportionally with delivered flow rate. One physiological study demonstrated a rise in intratracheal FiO_2_ from 67% to 93% as flow increased from 15 to 45 L·min^−1^ [13], supporting flows above 50 L·min^−1^ to maximize oxygenation. FiO_2_ should be set to 1.0, and patients should be instructed to breathe with their mouth closed to maintain the nasopharyngeal positive pressure generated by HFNO [52].

HFNO is generally well tolerated by awake patients, though some experience discomfort at flow rates of 50–70 L·min^−1^; in such cases, immediate flow reduction with subsequent titration after loss of consciousness is recommended [40]. A recent randomized trial in elective surgical patients identified 45 L·min^−1^ as the optimal pre-oxygenation flow rate, with higher flows offering limited additional extension of safe apnea time at the cost of increased discomfort [14]. In pediatric patients, flow is weight-based at 2 L·kg^−1^·min^−1^, with thresholds of 35 L·min^−1^ (0–15 kg), 40 L·min^−1^ (15–30 kg), and 50 L·min^−1^ (30–50 kg) [22,23].

The Lodenius trial randomized 80 adults undergoing rapid sequence induction to HFNO (THRIVE, 70 L·min^−1^, FiO_2_ 1.0) versus facemask pre-oxygenation [13] and demonstrated non-inferiority of HFNO for safe apnea time. In aggregate, HFNO matches or exceeds facemask pre-oxygenation in achieving the EtO_2_ target, with the unique advantage of remaining in situ throughout laryngoscopy—a property that becomes decisive in the apneic phase. A complementary perspective is provided by the PREOPTI-DAM trial, which randomised 186 patients with anticipated difficult airway management undergoing scheduled surgery to four minutes of pre-oxygenation with HFNO or facemask [53]. The primary composite outcome of desaturation ≤ 94% or need for bag-mask ventilation occurred in 2 of 95 patients (2%) with HFNO versus 7 of 90 (8%) with facemask (adjusted difference −5.6, 95% CI −11.8 to 0.6; *p* = 0.10), a non-significant result in a trial the authors acknowledged was underpowered, although patient-reported intubation experience was significantly better with HFNO. This trial is a useful corrective: in elective patients without marked baseline hypoxemia, the incremental benefit of HFNO over a well-conducted facemask technique may be small.

### 4.3. NIV vs. Conventional Facemask

In patients with significant baseline hypoxemia, conventional facemask pre-oxygenation is frequently insufficient to achieve an adequate EtO_2_ target, owing to atelectasis, intrapulmonary shunt, and the inability of passive oxygen delivery to recruit collapsed alveoli. NIV—by combining a sealed interface, controlled FiO_2_ up to 1.0, positive end-expiratory pressure (typically 5–10 cmH_2_O), and inspiratory pressure support—addresses each of these limitations simultaneously [42,43,44].

The landmark PREOXI trial randomised 1301 critically ill adults requiring emergency tracheal intubation to pre-oxygenation with NIV versus an oxygen mask [11]. The incidence of hypoxemia (SpO_2_ < 85%) between induction and two minutes after intubation was 9.1% with NIV versus 18.5% with the oxygen mask, an absolute risk reduction of 9.4 percentage points (95% CI −13.2 to −5.6; *p* < 0.001); cardiac arrest occurred in 1 of 645 patients (0.2%) versus 7 of 656 (1.1%), respectively. The effect was consistent across pre-specified subgroups and corresponds to approximately a halving of the relative risk of clinically significant desaturation. A secondary analysis examining effect modification by pulmonary comorbidity has been reported in abstract form [54]; as a conference abstract that has not yet undergone full peer review, it is noted here as hypothesis-generating and is not used to support any recommendation. These findings confirm and extend earlier work demonstrating the superiority of pressure-supported pre-oxygenation in hypoxemic critically ill patients [55].

Despite this robust evidence, NIV-based pre-oxygenation remains substantially under-implemented in intensive care units worldwide [56]. Operational barriers—equipment availability, staff familiarity, patient tolerance, and the time required to establish an effective interface seal—contribute to this implementation gap. In awake patients with respiratory distress, NIV may be poorly tolerated and may delay intubation in a deteriorating patient; clinical judgment regarding the threshold at which “pre-oxygenation optimization” must yield to “definitive airway securement” remains essential.

### 4.4. HFNO vs. NIV—And the Combined Approach

The most clinically consequential question in contemporary peri-intubation oxygenation is not “HFNO or facemask?” but “HFNO, NIV, or both?”. The systematic review and network meta-analysis by Pitre and colleagues is the most authoritative synthesis currently available [10]. It included 15 randomised trials enrolling 3420 critically ill adults requiring intubation in the intensive care or emergency department setting. Non-invasive positive-pressure ventilation (NIPPV) probably reduced the incidence of hypoxemia during intubation compared with HFNO (relative risk 0.73, 95% CI 0.55–0.98; moderate certainty) and reduced it compared with facemask oxygen (0.51, 0.39–0.65; high certainty), while HFNO reduced hypoxemia compared with facemask oxygen (0.69, 0.54–0.88; high certainty). NIPPV probably reduced serious adverse events versus facemask oxygen (0.30, 0.12–0.77; moderate certainty). Importantly, none of the strategies affected the rate of successful intubation at the first attempt or all-cause mortality. Two interpretive cautions follow. First, the demonstrated advantage of NIV over HFNO concerns the prevention of hypoxemia and of serious adverse events and should not be extended to outcomes for which no difference was demonstrated. Second, the treatment rankings produced by a network meta-analysis summarise average effects across trial populations; they do not constitute a validated clinical algorithm, and this analysis neither evaluated nor validated any PaO_2_/FiO_2_ threshold for choosing between modalities.

The only large randomised head-to-head comparison of NIV with HFNO for pre-oxygenation is the FLORALI-2 trial [57], which is central to the phenotype question and therefore merits detailed consideration. In 28 French intensive care units, 313 patients intubated for acute hypoxemic respiratory failure (PaO_2_/FiO_2_ ≤ 300 mmHg) were analysed in the intention-to-treat population (142 NIV, 171 HFNO), with randomisation stratified a priori by PaO_2_/FiO_2_ (≤200 versus > 200 mmHg). In the overall population the trial was negative: severe hypoxemia (SpO_2_ < 80%) occurred in 33 of 142 patients (23%) after pre-oxygenation with NIV and in 47 of 171 (27%) after HFNO (absolute difference −4.2%, 95% CI −13.7 to 5.5; *p* = 0.39). In the pre-specified stratum with moderate-to-severe hypoxemia (PaO_2_/FiO_2_ ≤ 200 mmHg; *n* = 242), severe hypoxemia was less frequent with NIV than with HFNO (28 of 117 [24%] versus 44 of 125 [35%]; adjusted odds ratio 0.56, 95% CI 0.32–0.99; *p* = 0.0459). This stratified subgroup signal is the principal empirical basis for the PaO_2_/FiO_2_ 200 mmHg threshold used descriptively throughout this review, and its limitations must be stated plainly: it arises within a trial whose primary outcome was neutral, the confidence interval approaches unity, and it has not been replicated in a dedicated confirmatory trial. A PaO_2_/FiO_2_ of 200 mmHg should therefore be regarded as a pragmatic descriptor of a more vulnerable phenotype, and as a reasonable stratification variable for future trials, rather than as a validated decision threshold.

Two important caveats refine this hierarchy. First, in non-hypoxemic or mildly hypoxemic patients, the incremental benefit of NIV over HFNO is attenuated, and the superior tolerability and operational simplicity of HFNO may favor its use [57]. Second, NIV alone fails to address the continuity-of-oxygenation problem during laryngoscopy: the sealed mask must be removed at induction, interrupting positive pressure and oxygen delivery at the most vulnerable moment.

This limitation is precisely what the combined NIV-plus-HFNO approach seeks to address. The OPTINIV trial randomised 49 hypoxemic ICU patients (25 to the combined strategy, 24 to NIV alone) to NIV alone versus NIV for pre-oxygenation plus HFNO maintained throughout the apneic interval [9]. The median lowest SpO_2_ during intubation was higher with the combined strategy (100% [IQR 95–100] versus 96% [IQR 92–99]; *p* = 0.029), without adverse effects. The physiological rationale is straightforward: NIV provides alveolar recruitment and active denitrogenation during pre-oxygenation, while HFNO—left in situ when the NIV mask is removed for laryngoscopy—delivers uninterrupted apneic oxygenation through the most dangerous interval. The strength of this evidence should not be overstated. OPTINIV was a single-centre trial of fewer than 50 patients using a physiological primary endpoint, and no trial has yet compared the combined approach with NIV alone for patient-important outcomes. The combined strategy is therefore best described as promising and physiologically coherent rather than established, and it is a leading candidate for a confirmatory multicentre trial.

**Figure 3:**
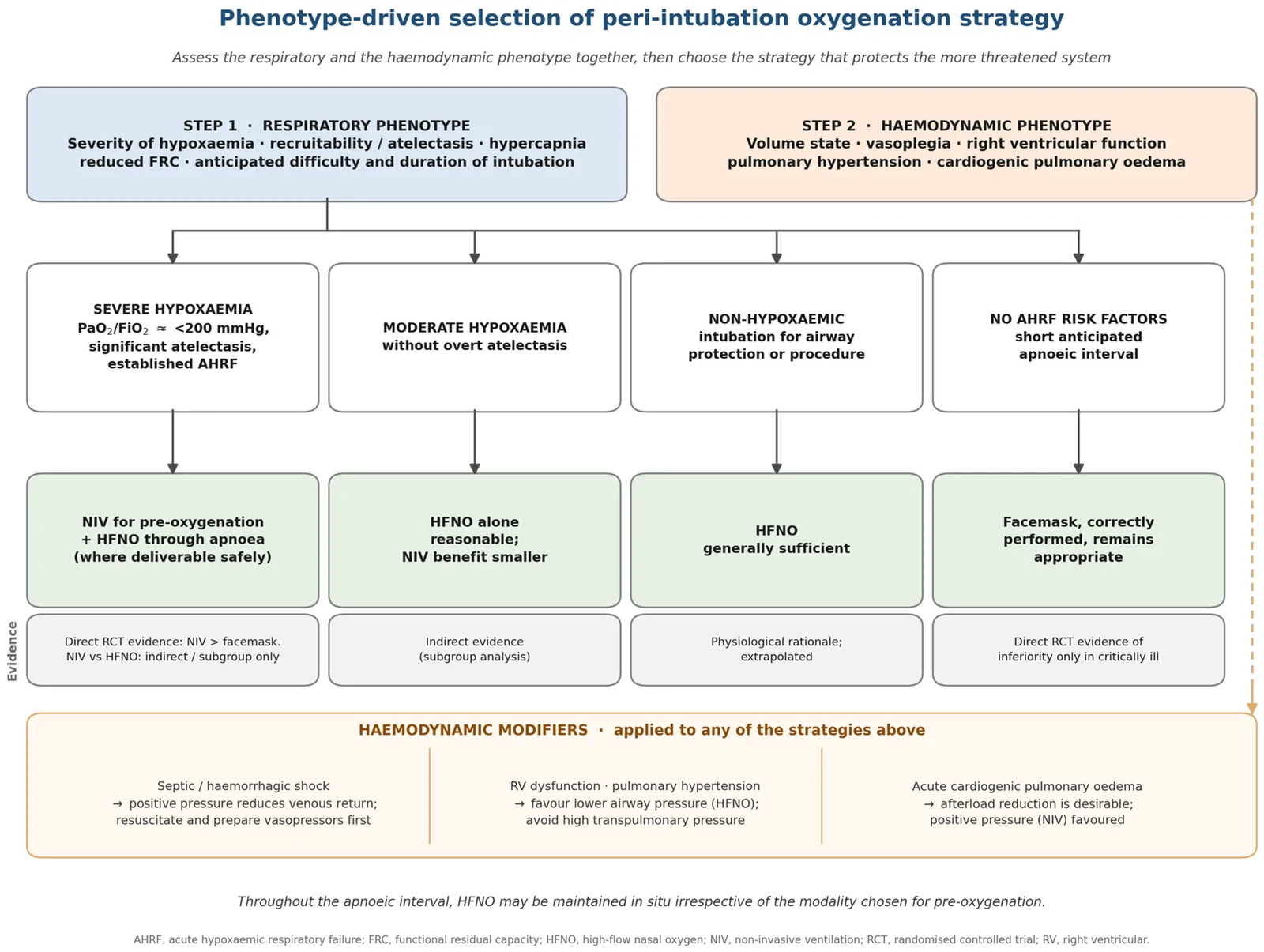
Phenotype-driven selection of the peri-intubation oxygenation strategy. The respiratory phenotype (severity of hypoxaemia, recruitability, hypercapnia, functional residual capacity, anticipated difficulty and duration of airway instrumentation) determines which modality is most likely to prevent desaturation, while the haemodynamic phenotype determines how safely that modality can be delivered. The grey band records the strength of the supporting evidence for each option; the lower panel lists haemodynamic modifiers that apply to any of the strategies. High-flow nasal oxygen may be maintained in situ throughout the apnoeic interval irrespective of the modality chosen for pre-oxygenation. The PaO_2_/FiO_2_ value of 200 mmHg is a pragmatic descriptor of a more vulnerable phenotype, not a validated decision threshold (see Section 4.4).

Taken together, these data support a phenotype-driven hierarchy, summarised in Figure 3 together with the haemodynamic modifiers discussed in Section 3.5 and an explicit indication of the strength of the evidence underlying each option:

In severely hypoxemic critically ill patients (PaO_2_/FiO_2_ below approximately 200 mmHg, significant atelectasis, or established AHRF), NIV is the modality most likely to prevent peri-intubation hypoxemia and is a reasonable first choice where it can be delivered safely, with HFNO maintained during the apneic phase when feasible. (Direct randomised evidence for NIV versus facemask; indirect and subgroup evidence for NIV versus HFNO; physiological rationale for the combination.)

In moderately hypoxemic patients without overt atelectasis, HFNO alone is a reasonable choice, offering uninterrupted oxygenation and superior tolerability, and the incremental benefit of NIV in this group appears smaller. (Indirect evidence, derived largely from subgroup analysis.)

In non-hypoxemic patients requiring intubation (for example, for airway protection or planned procedures), HFNO is generally sufficient and operationally simpler. (Physiological rationale and extrapolation from elective surgical populations.)

Conventional facemask pre-oxygenation, correctly performed, remains appropriate in patients without AHRF risk factors and with short anticipated apneic intervals. (Direct randomised evidence establishes its inferiority in critically ill patients but not in low-risk populations.)

It is useful to set this hierarchy against formal guideline recommendations, which are summarised in Table 3. The joint ESA/ESICM guideline on non-invasive respiratory support in the hypoxaemic perioperative and periprocedural patient issued 19 GRADE-based recommendations, of which the two graded 1B state that NIPPV or CPAP (according to local expertise) is preferred to conventional oxygen therapy for improving oxygenation and that NIPPV or CPAP should be considered immediately post-extubation in hypoxaemic patients at risk of respiratory failure after abdominal surgery [43]. The DAS/ICS/FICM/RCoA guidelines for the management of tracheal intubation in critically ill adults emphasise the primacy of oxygenation, including pre-oxygenation and peroxygenation, and recommend a modified rapid sequence approach [58]. The SIAARTI consensus addresses the obese patient specifically [59], and the OAA/DAS guidelines address obstetric airway management [60]. Two observations follow. First, no current guideline specifies a PaO_2_/FiO_2_ threshold at which NIV should replace HFNO; the hierarchy proposed here is an interpretive synthesis, not a guideline recommendation. Second, several of these documents predate PREOXI and the Pitre network meta-analysis, and forthcoming updates are likely to strengthen recommendations for positive-pressure pre-oxygenation in the critically ill. Institutional implementation, in any case, remains heterogeneous.

### 4.5. Pre-Oxygenation in Specific Populations

#### 4.5.1. Severe Obesity and Obstructive Sleep Apnea

A body mass index (BMI) greater than 35 kg·m^−2^ is an independent predictor of difficult mask ventilation and, in some cases, difficult intubation. Conventional facemask pre-oxygenation is of limited benefit in this population, as obesity-related pathophysiological changes substantially reduce safe apnea time [50,59]. Respiratory system compliance is reduced by up to 35%, FRC and expiratory reserve volume are diminished, diaphragmatic mechanical efficiency is approximately halved, and metabolic oxygen demand is increased [50,59,61].

Appropriate patient positioning during pre-oxygenation is essential. Elevating the head to a 25-degree incline with optimized airway alignment—the “ramped” position—is strongly advisable in this population and should be regarded as part of the standard technique rather than an optional refinement [59]. The optimal pre-oxygenation strategy is positive airway pressure, ideally with pressure support [59]. When NIV is unavailable or poorly tolerated, HFNO can be used as a standalone alternative or as an adjunct during facemask pre-oxygenation to compensate for mask leaks (FiO_2_ 1.0, 60 L·min^−1^, mouth closed).

Wu and colleagues compared HFNO versus facemask in 80 obese patients undergoing elective tracheal intubation, demonstrating higher PaO_2_ and significantly fewer peri-intubation desaturation events with HFNO [15]. Schutzer-Weissmann and colleagues demonstrated extension of safe apnea time to 18 min without desaturation in morbidly obese patients pre-oxygenated with HFNO [16]. Heinrich and colleagues, in patients undergoing bariatric surgery, showed that HFNO at FiO_2_ 1.0 and 50 L·min^−1^ significantly improved PaO_2_ at 5 and 7 min after rapid sequence induction compared to facemask, with non-inferiority versus CPAP at 7 cmH_2_O [17]. A recent systematic review tempers the magnitude of benefit when desaturation below 92% is the chosen outcome [10], reminding us that comparator choice and outcome definition substantially influence apparent effect size.

In OSA specifically, HFNO at flow rates >35 L·min^−1^ generates a positive nasal end-expiratory pressure of 3–5 mmHg, sufficient to prevent upper airway collapse during sedation and apnea [35]. Recent data confirm that HFNO maintained in situ following induction prolongs safe apnea time and reduces the incidence of desaturation in this population [35,62].

#### 4.5.2. High-Risk Obstetric Patients

Pregnancy is a state of physiological vulnerability to peri-intubation desaturation, with oxygen consumption increased by approximately 20% and FRC reduced by 20% as a consequence of diaphragmatic elevation by the gravid uterus [18,62]. These changes shorten the time to desaturation to 2–3 min of apnea, compared with 7–10 min in non-pregnant adults [18,62]. The clinical phenotype becomes one of frank AHRF risk in well-defined obstetric subgroups: pre-eclampsia complicated by pulmonary edema, peripartum cardiomyopathy with decompensation, amniotic fluid embolism, severe maternal sepsis, and respiratory viral infections in pregnancy. Concurrent obesity and OSA—increasingly prevalent—further compound the risk [35,62].

Zhou and colleagues demonstrated that HFNO maintains superior maternal oxygenation during rapid sequence induction compared to facemask pre-oxygenation [18]. The generation of positive nasopharyngeal pressure without the need for mask removal allows for continuous oxygenation throughout airway assessment and instrumentation, an advantage of particular value in the obstetric airway, where Mallampati progression, mucosal edema, and aspiration risk complicate management [60]. Current OAA/DAS guidance suggests the use of HFNO or low-flow nasal cannulae during the pre-intubation phase, acknowledging that the supporting evidence base remains limited [60]. A recent systematic review including 11 studies concluded that the perioperative advantages of HFNO over conventional oxygen therapy in the obstetric population remain uncertain [19], underscoring the need for adequately powered trials in this specific setting—particularly in obstetric patients meeting AHRF criteria, in whom the physiological rationale is strongest.

#### 4.5.3. Critically Ill Patients

Critically ill patients requiring emergency intubation represent the population in whom the evidence on pre-oxygenation modalities is most mature and most directly applicable. The INTUBE cohort documented peri-intubation cardiovascular complications in over 40% and severe hypoxemia in 9% of critically ill adults [1], with the risk further amplified in obese critically ill patients [3]. Optimal pre-oxygenation in this setting requires prior hemodynamic optimization, thorough team preparation, and selection of a pressure-supported modality whenever feasible [4,44].

The evidence reviewed in Section 4.3 and Section 4.4—PREOXI for the superiority of NIV over facemask [11], the Pitre network meta-analysis for the relative positioning of the three modalities [10], and FLORALI-2 for the direct NIV-versus-HFNO comparison [57]—applies in its strongest form to this population. On present evidence, NIV is the modality most likely to prevent peri-intubation hypoxemia in severely hypoxemic critically ill patients and is a reasonable default where it can be delivered safely; HFNO is appropriate when NIV is contraindicated or poorly tolerated, and in patients who are not severely hypoxemic; and the combined NIV-plus-HFNO approach is physiologically attractive but remains supported by a single small trial [9]. Persistent under-implementation of NIV-based pre-oxygenation in ICUs worldwide [56] represents a quality-of-care gap that the present body of evidence should help to close.

## 5. Apneic Oxygenation

### 5.1. Physiological Basis and Clinical Application

Apneic oxygenation refers to the continuous delivery of oxygen during the interval between induction of anesthesia and successful tracheal intubation, when the patient’s respiratory drive and neuromuscular activity are suppressed. Its objective is to maintain arterial oxygenation and delay desaturation, particularly in patients with predictors of difficult airway management, reduced functional residual capacity, increased metabolic oxygen demand, or established or impending acute hypoxemic respiratory failure [63,64].

**Figure 4:**
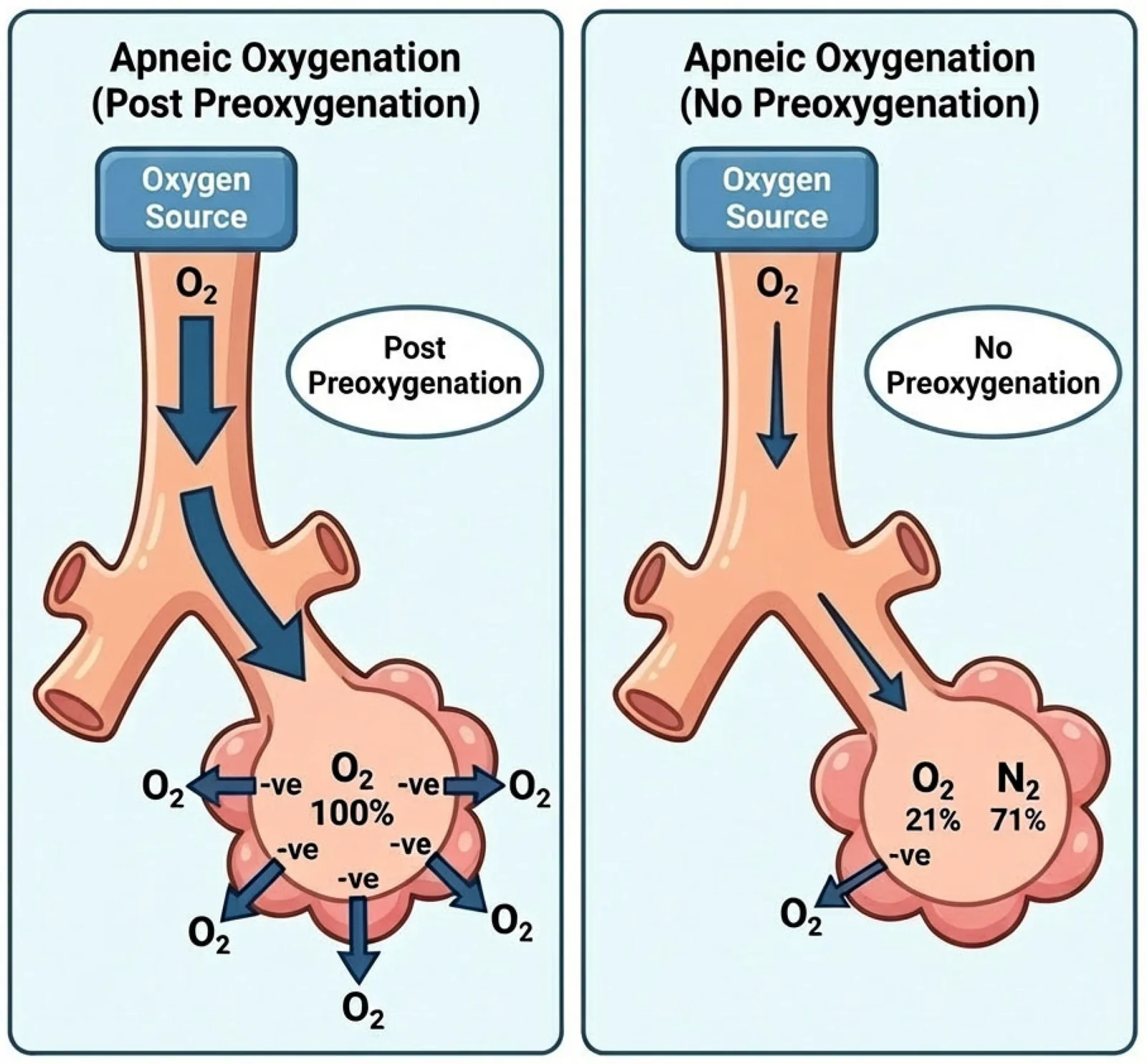
Physiological basis of apneic oxygenation and the role of pre-oxygenation. Left panel (post pre-oxygenation): after adequate denitrogenation the alveolus is filled with approximately 100% oxygen; the continued diffusion of oxygen into the pulmonary capillary blood generates a sub-atmospheric alveolar pressure that draws oxygen down the airway (aventilatory mass flow), sustaining arterial oxygenation during apnea. Right panel (no pre-oxygenation): residual alveolar nitrogen (N_2_ ≈ 71%) limits the alveolar oxygen fraction (≈21%) and markedly reduces the oxygen available for apneic uptake, resulting in rapid desaturation.

The physiological basis of apneic oxygenation is the principle of apneic mass-flow diffusion. During apnea, oxygen accumulated in the nasopharynx, oropharynx, and alveolar space continues to diffuse passively across the alveolar–capillary membrane into the bloodstream at a rate of approximately 250 mL·min^−1^. This net oxygen uptake generates a sub-atmospheric alveolar pressure, creating a sustained pressure gradient from the upper airways toward the alveoli that supports passive oxygen delivery in the absence of active ventilation [63,64] (Figure 4). Maintenance of upper airway patency is a prerequisite for this mechanism to operate and may be achieved through jaw thrust, mandibular subluxation, or nasopharyngeal airway placement.

Oxygen may be delivered during apnea via conventional low-flow devices (nasal cannulae or nasopharyngeal catheters at 3–10 L·min^−1^) or via HFNO at 30–70 L·min^−1^. HFNO offers three advantages of direct relevance to peri-intubation oxygenation in patients at risk of AHRF: substantially greater extension of safe apnea time, low-level positive nasopharyngeal pressure that opposes airway collapse and atelectasis, and partial CO_2_ washout from the upper airway dead space [63,64]. The technique of transnasal humidified rapid-insufflation ventilatory exchange (THRIVE), originally described by Patel and Nouraei, formalizes HFNO at 70 L·min^−1^ with FiO_2_ 1.0, initiated ten minutes before induction and maintained without interruption until airway securement [20]. In their original cohort, which included patients with anatomically and physiologically difficult airways, THRIVE yielded a mean apnea time of 14 min (median 9–19, range 5–65) without episodes of desaturation below 90% or clinically significant CO_2_-related adverse events; the EtCO_2_ increase rate was 0.15 kPa·min^−1^ [20].

The clinical relevance of apneic oxygenation in the broader peri-intubation context is supported by the OPTIMASK international study, which compared facemask alone versus combined facemask plus HFNO during pre-oxygenation, with HFNO maintained through the apneic phase until intubation [24]. In 450 patients undergoing general anesthesia regardless of BMI, end-tidal oxygen values at the time of intubation were significantly higher in the combined facemask-plus-HFNO group, confirming the additive benefit of maintaining HFNO through the apneic interval—a principle that becomes particularly valuable when the patient has reduced physiological reserve.

### 5.2. Apneic Oxygenation in Severe Obesity and OSA

Severe obesity and OSA represent the populations in whom the benefit of apneic oxygenation with HFNO is most consistently demonstrated. The mechanisms are physiologically intertwined: reduced FRC limits the alveolar oxygen reservoir, increased metabolic demand accelerates oxygen consumption, and predisposition to upper airway collapse during sedation compromises the patency required for passive oxygen diffusion. HFNO addresses all three issues simultaneously—by maintaining a high alveolar FiO_2_, generating positive nasopharyngeal pressure that stents the upper airway, and remaining in situ during airway instrumentation.

Wong and colleagues randomized morbidly obese patients (BMI > 40 kg·m^−2^) to HFNO versus conventional oxygen therapy during apnea and demonstrated a significant extension of safe apnea time to 4.3 min versus 1.3 min in the control group [21]. Schutzer-Weissmann and colleagues extended this signal to 18 min of safe apnea in morbidly obese patients pre-oxygenated and maintained on HFNO [16]. In OSA, oxygen flows exceeding 20 L·min^−1^ reduce the apnea–hypopnea index by 64%, and flows exceeding 35 L·min^−1^ generate sufficient positive nasal end-expiratory pressure (3–5 mmHg) to prevent upper airway collapse [35]—a mechanism that translates directly into preserved upper airway patency during the sedated apneic interval.

Taken together, these data support HFNO-based apneic oxygenation as a reasonable first-line approach in obese patients undergoing intubation when AHRF risk is present, with NIV used for pre-oxygenation in the most severely hypoxemic subset and removed at laryngoscopy in favour of continued HFNO. This recommendation should be framed carefully. Obesity identifies a population with reduced oxygen reserve, in whom optimisation of pre-oxygenation and positioning is particularly important; it does not by itself establish the superiority of one modality over another. No adequately powered trial has compared NIV with HFNO for pre-oxygenation using patient-important outcomes in this phenotype, and the available comparative evidence is insufficient to establish NIV as universally superior to HFNO in obese patients.

### 5.3. Apneic Oxygenation in the Pediatric Patient

Children desaturate substantially faster than adults following cessation of ventilation, owing to reduced FRC, higher mass-specific oxygen consumption, and predisposition to upper airway collapse [65]. Mean time to desaturation below 90% SpO_2_ has been documented at 160 s in children, 382 s in adolescents, and as short as 97 s in neonates, with desaturation episodes occurring in 4–10% of pediatric inductions and up to 20% during endotracheal intubation [65]. These figures indicate that the physiological margin for error is narrow in children and that many—though by no means all—pediatric intubations share features of the AHRF-risk phenotype; the framework discussed here is therefore most applicable to children with reduced reserve or pre-existing hypoxemia rather than to every pediatric intubation.

Humphreys and colleagues randomized 48 children under ten years of age with normal airways to HFNO versus facemask pre-oxygenation; HFNO was delivered at 2 L·kg^−1^·min^−1^ with flow rates of 35, 40, or 50 L·min^−1^ stratified by weight [22]. Safe apnea time with SpO_2_ > 92% was doubled in the HFNO group, reaching approximately ten minutes [22,23]. Beyond this threshold, progressive hypercapnia becomes the dominant limiting factor: in pediatric patients weighing 10–20 kg, transcutaneous PCO_2_ rises at approximately 0.55 kPa·min^−1^, substantially faster than in adults [23,66]. For this reason, HFNO-based apneic oxygenation in children is considered safe for procedures with anticipated apneic intervals of 5–6 min, beyond which continuous CO_2_ monitoring is mandatory and clinical reassessment is required.

### 5.4. Limitations: Progressive Hypercapnia and the Chronic CO_2_-Retainer

The principal limitation of apneic oxygenation—whether delivered via HFNO or conventional devices—is progressive hypercapnia. In healthy adults during apnea, PaCO_2_ rises at approximately 3 mmHg·min^−1^ until a plateau of around 65 mmHg [67]. High-flow HFNO (up to 70 L·min^−1^) attenuates CO_2_ accumulation through enhanced dead-space washout—the EtCO_2_ increase rate during apneic HFNO oxygenation in adults is 0.12–0.17 kPa·min^−1^, compared with a minimal 0.03 kPa·min^−1^ during spontaneous breathing under HFNO [68,69]—but does not eliminate it. The precise mechanisms of CO_2_ clearance during HFNO remain incompletely understood; cardiogenic oscillations, gas mixing within the anatomical dead space, and micro-ventilation induced by pharyngeal pressure variations have been proposed [70].

The healthy-adult figure of 3 mmHg·min^−1^ cannot be assumed to apply uniformly across all patient phenotypes. Patients with severe COPD, chronic hypercapnic respiratory failure, advanced obesity-hypoventilation syndrome, or neuromuscular disease exhibit substantially faster CO_2_ accumulation, lower buffering reserves, and reduced tolerance to acute hypercapnia. In these populations, even modest CO_2_ rises may produce clinically significant acidosis, with consequent impairment of myocardial contractility, pulmonary vasoconstriction, and altered cerebral hemodynamics. Subgroup-specific safety thresholds have not been formally established, and conservative apneic time limits with continuous transcutaneous or end-tidal CO_2_ monitoring should be applied in patients with chronic CO_2_ retention or limited buffering capacity. Where prolonged apnea is anticipated, the threshold for definitive airway securement should be lowered accordingly.

The theoretical risk of gastric insufflation during HFNO has not translated into clinically significant complications in the published literature. The positive nasopharyngeal pressure generated by HFNO with the patient’s mouth closed remains well below the lower esophageal sphincter opening pressure, and no clinically meaningful events—including regurgitation or pulmonary aspiration of gastric contents—have been reported in association with HFNO use, even in severely obese patients. A recent systematic review found no increase in gastric volume attributable to HFNO, though the supporting evidence was graded as low certainty [71].

## 6. HFNO During Awake Tracheal Intubation

Awake tracheal intubation (ATI) is the gold-standard technique when difficult mask ventilation and difficult intubation are simultaneously anticipated. More recently, the concept of the physiologically difficult airway has broadened the indications for ATI beyond purely anatomical considerations, positioning it as a clinically appropriate alternative to rapid sequence intubation in hemodynamically and respiratorily fragile patients [5,36]. In patients at risk of AHRF, the cardiovascular consequences of standard induction agents—vasodilation, myocardial depression, loss of sympathetic tone—may precipitate circulatory collapse superimposed on already marginal oxygen delivery, while the abolition of spontaneous respiratory drive removes the patient’s residual capacity for compensatory ventilation. ATI preserves both spontaneous ventilation and cardiovascular tone during airway instrumentation, and is therefore of direct relevance to several high-risk phenotypes covered in this review: severely hypoxemic critically ill patients with marginal hemodynamic reserve, patients with morbid obesity or advanced OSA, patients with critically reduced respiratory reserve from chronic cardio-respiratory disease, and selected high-risk obstetric patients [62,72].

HFNO at flow rates of 40–70 L·min^−1^ has been widely adopted as a supportive technique during ATI, providing concurrent benefits across multiple physiological domains [25,73]. First, continuous delivery of high-flow humidified oxygen at FiO_2_ 1.0 maintains arterial oxygenation throughout the procedure, providing a meaningful safety margin during a technically demanding and often prolonged bronchoscopic or videolaryngoscopic passage—a property of particular value in patients with reduced FRC and shortened safe apnea time. Second, the positive nasopharyngeal pressure generated by high-flow delivery partially stents the upper airway, attenuating the pharyngeal collapse that may accompany topicalization-induced loss of muscle tone or light sedation. Third, thermal conditioning of inspired gas—warmed to 37 °C and fully humidified—reduces mucosal drying and irritation caused by prolonged mouth breathing during procedural preparation, improving patient comfort and reducing the perception of dyspnea [25,73]. The combined effect is a more cooperative patient and an extended margin of safety during a procedure in which time pressure must not be allowed to compromise technical care.

From a practical standpoint, the nasal cannulae used for HFNO delivery do not impede passage of a flexible bronchoscope through either the nasal or the oral route, and the two devices can be used simultaneously without mutual interference. Asymmetric nasal cannula designs, in which one prong is shorter or absent, are available and may further optimize access. When ATI is performed using a videolaryngoscope rather than a flexible bronchoscope, the benefits of HFNO are fully preserved, as the cannulae remain in situ and continue to deliver oxygenation and positive nasopharyngeal pressure throughout laryngoscopy and tube passage [25,73].

A relevant consideration in the AHRF-risk patient is the threshold for converting from ATI to rapid airway securement. ATI is, by design, a careful and unhurried technique. If the patient deteriorates during the procedure—progressive desaturation despite HFNO at maximal settings, hemodynamic instability, or loss of cooperation—the operator must be prepared to abandon the awake approach in favor of definitive airway securement. The presence of HFNO during this transition is itself an advantage, as it provides uninterrupted oxygenation through the conversion and may extend the operator’s decision window. In this sense, HFNO during ATI functions not only as a supportive technique but as a physiological safety net during a procedure with intrinsically high stakes.

## 7. Emergency and Trauma Settings

The principles of peri-intubation oxygenation developed in the preceding sections find their most demanding application in the emergency and trauma context, where hemodynamic instability, potentially challenging airway anatomy, and extreme time pressure converge to create conditions of maximal procedural risk [5,36]. In trauma patients requiring rapid sequence intubation, the concept of the physiologically difficult airway [5] is particularly relevant: the goal of securing the airway must be pursued while preserving—or at minimum not further compromising—an already tenuous cardiovascular and respiratory equilibrium. The peri-intubation hypoxemia, hemodynamic collapse, and cardiac arrest rates documented by the INTUBE study in mixed critically ill populations [1] are typically equaled or exceeded in trauma cohorts, particularly in those with hemorrhagic shock, thoracic injury, or traumatic brain injury.

### 7.1. Physiological Rationale for HFNO and NIV in the Emergency Setting

In this context, HFNO offers several simultaneous physiological benefits. It maintains adequate oxygen delivery in patients with already reduced oxygen-carrying capacity from blood loss or anemia, provides continuous oxygenation during laryngoscopy without requiring mask removal [74,75], generates positive airway pressure that supports alveolar recruitment in patients with pulmonary contusion or atelectasis from supine positioning [31,39], and extends safe apnea time even in the presence of hypovolemia [50]. In traumatic brain injury, where strict avoidance of hypoxemia is critical to prevent secondary brain injury and where blood pressure targets must be maintained to preserve cerebral perfusion pressure, even brief desaturation episodes during intubation may have lasting neurological consequences—making uninterrupted oxygenation a priority that HFNO is well positioned to deliver. In thoracic trauma complicated by pulmonary contusion, maintaining oxygenation while minimizing ventilation–perfusion mismatch before definitive airway securement represents a particular challenge to which HFNO contributes meaningfully [5].

The role of NIV in the emergency setting is more constrained than in the controlled ICU environment. NIV requires a degree of patient cooperation, an adequate mask seal, and time to titrate pressures—all of which may be unavailable in agitated, intoxicated, or anatomically distorted trauma patients. Where time and patient condition permit, NIV-based pre-oxygenation followed by HFNO maintained during apnea remains the physiologically optimal strategy. In the more frequent scenario of urgent intubation in a poorly cooperating patient, HFNO is often the only practicable non-invasive modality and should be preferred to facemask alone whenever logistics allow.

### 7.2. Clinical Evidence

Raineri and colleagues evaluated the efficacy and safety of HFNO in 45 patients undergoing rapid sequence intubation for urgent abdominal surgery, recording a significant increase in oxygen saturation at all time points compared to baseline, with a minimum SpO_2_ of 96%, a maximum apnea time of 12 min, and an EtCO_2_ at the time of intubation of 36 mmHg [26]. A recent systematic review and meta-analysis by Tang and colleagues pooled trials of HFNO versus facemask for pre- and apneic oxygenation during rapid sequence induction in emergency surgery, concluding that HFNO is superior to facemask in terms of oxygenation maintenance during the peri-intubation period [27]. The Cochrane review by White and colleagues synthesized evidence on apneic oxygenation across emergency department, ICU, prehospital, and operating theatre settings, finding heterogeneous evidence with a benefit signal in selected high-risk groups [28]. A large multicentre trial (Pre-AeRATE) is currently evaluating the safety and efficacy of HFNO for pre-oxygenation and apneic oxygenation during emergency rapid sequence intubation in the emergency department [29], and its results are expected to clarify the evidence base specifically for this setting.

### 7.3. Practical Considerations in Trauma Airway Management

Several practical considerations are specific to trauma and emergency airway management. The nasal interface used for HFNO does not require cervical manipulation, facilitating oxygenation in patients requiring manual inline stabilization of the cervical spine—a meaningful operational advantage over facemask techniques that may interfere with collar positioning. Unlike NIV masks, HFNO does not need to be removed before laryngoscopy, eliminating any interruption in oxygen delivery at the most critical moment of the procedure. In hemorrhagic shock, the maintenance of oxygen delivery assumes heightened importance given the reduction in oxygen-carrying capacity imposed by blood loss; in this setting, every minute of preserved arterial oxygenation contributes to maintaining adequate tissue oxygen flux while resuscitation proceeds. In polytrauma scenarios, the hands-free, continuous nature of HFNO delivery is operationally advantageous when the clinical team is simultaneously managing multiple life-threatening injuries [5].

While dedicated randomized trials in trauma populations remain limited, the physiological rationale is compelling, extrapolation from the emergency department intubation literature [29] and from critically ill ICU cohorts [74,75,76] supports the consideration of HFNO as a valuable adjunct within trauma airway management protocols, and the forthcoming Pre-AeRATE results [29] will provide trial-level evidence specific to the emergency setting.

## 8. Disease-Specific Considerations in Patients at Risk of AHRF

The peri-intubation management of patients with chronic cardio-respiratory disease differs in clinically meaningful ways from that of physiologically intact adults, and the choice of oxygenation strategy must be informed by the specific pathophysiology of the underlying condition. The available evidence specifically addressing peri-intubation HFNO and NIV in each of these populations remains limited; the considerations below combine direct evidence (where available) with extrapolation from acute-care studies and physiological reasoning. This evidentiary asymmetry is itself an important conclusion and informs the research priorities identified in this paper.

### 8.1. Chronic Obstructive Pulmonary Disease

COPD is among the most common comorbidities in patients requiring emergency or urgent intubation. Peri-intubation challenges in COPD include impaired gas exchange, dynamic hyperinflation, intrinsic PEEP, increased work of breathing, mucus hypersecretion, and—in advanced disease—chronic hypercapnia with blunted central chemoreceptor responsiveness.

The physiological profile of HFNO is well aligned with several of these features: dead-space CO_2_ washout may help offset increased ventilatory demand, low-level positive airway pressure can counterbalance intrinsic PEEP, heated humidification supports mucociliary clearance, and reduced inspiratory work of breathing is particularly beneficial in patients with limited respiratory reserve. Acute-care data demonstrate benefit of HFNO in selected COPD patients, particularly in mild-to-moderate hypercapnic respiratory failure where it may serve as an alternative to NIV in patients intolerant of mask interfaces [38]. In severe hypercapnic exacerbation, however, NIV remains the reference modality [43], and its substitution with HFNO is not appropriate.

Practical considerations:

HFNO is a reasonable pre-oxygenation strategy in patients with mild-to-moderate disease and no significant chronic CO_2_ retention.

In chronic hypercapnic respiratory failure, NIV with pressure support is the preferred pre-oxygenation modality; HFNO may be maintained during apnea to provide uninterrupted oxygenation.

Apneic oxygenation time must be conservatively limited; the 3 mmHg·min^−1^ assumption from healthy adults underestimates clinically significant acidosis in patients with chronic hypercapnia [67].

Continuous transcutaneous or end-tidal CO_2_ monitoring is strongly recommended.

### 8.2. Heart Failure

Patients with heart failure—particularly those with reduced ejection fraction, pulmonary hypertension, or pulmonary congestion—face heightened peri-intubation risk of hypoxemia, hemodynamic collapse, and arrhythmia. Pre-existing diaphragmatic dysfunction and pulmonary edema reduce FRC and gas exchange efficiency, while elevated left atrial pressure increases susceptibility to alveolar flooding under any peri-intubation respiratory stressor. The induction phase itself imposes substantial hemodynamic challenge: sympatholysis, vasodilation, and the shift from spontaneous (negative-pressure) to positive-pressure ventilation can precipitate acute decompensation.

HFNO confers physiological benefits relevant to this population: a reduction in work of breathing decreases myocardial oxygen demand; low-level positive airway pressure may modestly reduce left ventricular preload and afterload through mechanisms analogous to (although substantially weaker than) those of CPAP; and uninterrupted humidified oxygen delivery prevents desaturation that would worsen myocardial ischemia. In acute decompensated heart failure with pulmonary edema, however, NIV (CPAP or BiPAP) remains the reference modality for both pre-oxygenation and acute respiratory support, owing to the magnitude of positive airway pressure required to reverse alveolar flooding.

Practical considerations:

HFNO is appropriate in stable, compensated heart failure across pre-oxygenation and apneic phases.

In acute decompensated heart failure with pulmonary edema, NIV is the preferred pre-oxygenation modality; the combined approach (NIV → HFNO during apnea) is particularly attractive.

Hemodynamic monitoring should be tailored to the severity of cardiac dysfunction; HFNO is hemodynamically well tolerated.

Awake tracheal intubation deserves consideration in the most fragile patients, in whom the cardiovascular consequences of induction may be intolerable.

### 8.3. Interstitial Lung Disease

ILD encompasses a heterogeneous group of disorders characterized by reduced lung compliance, diffusion impairment, and—in advanced disease—severe restrictive physiology with markedly reduced FRC and gas exchange surface. The peri-intubation phase in ILD is particularly demanding because the margin for desaturation is narrow, alveolar recruitment maneuvers are limited by reduced compliance and high airway pressures, and the risk of barotrauma during subsequent mechanical ventilation is elevated.

HFNO does not address the underlying restrictive defect, but its capacity to deliver high FiO_2_ with minimal patient effort, generate low-level positive airway pressure, and avoid mask-related discomfort makes it an attractive component of peri-intubation respiratory support. Direct peri-intubation evidence specific to ILD is sparse; reasoning is therefore largely physiological and extrapolated from acute respiratory failure trials in which ILD patients constituted a subgroup.

Practical considerations:

Pre-oxygenation with HFNO at FiO_2_ 1.0 and 50–70 L·min^−1^ is reasonable in mild-to-moderate disease; in advanced disease the benefit is limited by the restrictive defect itself.

In severely hypoxemic ILD patients, NIV-based pre-oxygenation is preferred, with HFNO maintained during apnea.

Apneic time should be conservatively limited; rapid desaturation should be anticipated, with a low threshold for definitive airway securement.

Awake tracheal intubation is a reasonable strategy in patients with advanced ILD and limited reserve, where the cardiopulmonary consequences of induction may be poorly tolerated.

### 8.4. Severe Obesity and Obstructive Sleep Apnea

Severe obesity and OSA are the most extensively studied populations in the peri-intubation HFNO literature and are discussed in detail in Section 4.5.1 and Section 5.2. The principal physiological substrate for benefit is the combination of reduced FRC, accelerated desaturation during apnea, and predisposition to upper airway collapse with sedation. The supporting evidence base—Wu [15], Schutzer-Weissmann [16], Heinrich [17], Wong [21], and the OSA mechanistic data of McGinley [35]—consistently demonstrates an extension of safe apnea time and a reduction in desaturation events with HFNO compared with facemask, with non-inferiority versus CPAP in selected populations.

Two considerations specific to this section deserve emphasis. First, patients with concurrent obesity-hypoventilation syndrome (OHS) must be regarded as hypercapnia-prone and managed according to the principles outlined for chronic CO_2_ retention in Section 8.1: conservative apneic time limits, continuous CO_2_ monitoring, and a preference for NIV-based pre-oxygenation when feasible. The frequent overlap of severe obesity, OSA, and OHS in the same patient warrants explicit pre-procedural assessment of baseline arterial blood gases or capnography whenever possible. Second, the ramped position (25-degree head elevation with optimized airway alignment) is mandatory in this population regardless of the oxygenation modality chosen, and its omission is among the most preventable causes of peri-intubation desaturation in obese patients [59].

## 9. Monitoring Considerations

Effective peri-intubation oxygenation cannot be dissociated from appropriate monitoring. In patients at risk of AHRF, monitoring serves two distinct but interconnected purposes: early detection of clinical deterioration—particularly hypoxemia, hypercapnia, and hemodynamic instability—and physiological titration of the chosen oxygenation strategy to the individual patient’s response. The standard of care during peri-intubation management includes continuous pulse oximetry, electrocardiography, non-invasive blood pressure or invasive arterial pressure monitoring, and end-tidal capnography once the airway is secured. Beyond these established parameters, several adjunctive modalities deserve consideration in the AHRF-risk setting.

### 9.1. CO_2_ Monitoring During Apneic Oxygenation

The principal physiological limitation of apneic oxygenation—progressive hypercapnia—has been discussed in Section 5.4. Continuous CO_2_ monitoring during the apneic phase is therefore not optional but mandatory in selected populations: pediatric patients, in whom CO_2_ accumulation is approximately twofold faster than in adults [23,66]; patients with chronic hypercapnic respiratory failure (severe COPD, OHS, neuromuscular disease), in whom baseline buffering capacity is reduced; and any patient in whom prolonged apnea is anticipated.

Transcutaneous CO_2_ monitoring (TcCO_2_) is particularly well suited to this context, as it provides continuous, non-invasive PaCO_2_ estimation without requiring an artificial airway and is unaffected by the gas dilution issues that limit conventional end-tidal capnography during HFNO use [66,77]. Dedicated nasal cannulae capable of EtCO_2_ sampling during HFNO have also been developed and offer a practical alternative where TcCO_2_ is unavailable [66]. Both modalities should be considered standard equipment in centres performing prolonged apneic oxygenation, awake tracheal intubation, or peri-intubation management of high-risk AHRF phenotypes.

### 9.2. Electrical Impedance Tomography

Electrical impedance tomography (EIT) is a non-invasive, radiation-free bedside imaging modality that provides real-time visualization of regional ventilation distribution and changes in end-expiratory lung volume (EELV). Its clinical relevance to peri-intubation oxygenation is twofold. First, EIT allows for direct visualization of the alveolar recruitment effect of HFNO and NIV—Riera and colleagues demonstrated using EIT that HFNO increases EELV in a dose-dependent manner [32], providing mechanistic confirmation of the low-level positive-pressure effect inferred from earlier studies. Second, EIT may inform individualized titration of pressure and flow settings in patients with heterogeneous lung disease, where regional ventilation distribution cannot be predicted from global oxygenation indices alone.

While EIT is not yet a routine component of peri-intubation monitoring, its incorporation into clinical research protocols is rapidly expanding, and emerging data support its utility as a physiology-informed tool for tailoring oxygenation strategies to the individual patient—particularly in critically ill, ILD, and severely obese populations, in whom uniform application of standard settings is unlikely to be optimal. Future trials examining HFNO versus NIV versus combined approaches in well-defined AHRF phenotypes would benefit substantially from EIT-based outcome assessment.

### 9.3. Emerging Adjunctive Modalities

Several investigational tools warrant brief mention. Diaphragmatic ultrasonography provides a non-invasive, bedside assessment of diaphragmatic function and effort, with potential applications in identifying patients in whom inspiratory unloading by HFNO or NIV is most likely to translate into clinical benefit. Respiratory rate variability analysis and pulse oximetry waveform analysis are emerging techniques for detecting subtle early changes in respiratory drive or work of breathing that may precede overt desaturation. Esophageal pressure monitoring, although invasive and not routinely available, allows for direct quantification of inspiratory effort and transpulmonary pressure and has been proposed as a research tool for personalized titration of non-invasive support.

The integration of these modalities into standard peri-intubation practice is not currently supported by sufficient evidence to justify routine adoption. However, their inclusion in clinical research protocols represents a scientifically valuable direction. Prospective studies combining these tools with HFNO, NIV, or the combined approach may help define optimal modality selection and titration in specific patient phenotypes, ultimately enabling a more personalized and physiologically targeted approach to peri-intubation oxygenation in patients at risk of AHRF.

## 10. Conclusions

Tracheal intubation in patients at risk of acute hypoxemic respiratory failure remains a high-stakes procedure, in which even brief desaturation may precipitate cardiovascular collapse, hypoxic brain injury, or death. The peri-intubation interval—from pre-oxygenation, through induction and apnea, to successful airway securement—is the critical window in which non-invasive oxygenation strategies must operate. Within this window, three modalities define the contemporary therapeutic landscape: high-flow nasal oxygen (HFNO), non-invasive ventilation (NIV/NIPPV), and their combination.

The available evidence supports a phenotype-driven hierarchy rather than a single dominant strategy. In severely hypoxemic critically ill patients with established or impending AHRF, NIV is the modality most likely to prevent peri-intubation hypoxemia and is a reasonable first choice where it can be delivered safely, with HFNO ideally maintained throughout the apneic interval to preserve continuity of oxygenation during laryngoscopy (the combined OPTINIV approach, which remains promising rather than established). In moderately hypoxemic patients without significant atelectasis, HFNO alone offers an attractive balance of effectiveness, tolerability, and operational simplicity. In non-hypoxemic patients requiring intubation for airway protection or planned procedures, HFNO is generally sufficient. Conventional facemask pre-oxygenation, while still appropriate in patients without AHRF risk factors, is increasingly difficult to defend as a default in this population. Throughout, the strength of the supporting evidence varies considerably: it is strongest for the superiority of positive-pressure pre-oxygenation over facemask oxygen in unselected critically ill adults and substantially weaker—resting on subgroup analyses, extrapolation, or physiological reasoning—for the finer distinctions between modalities within specific disease phenotypes. The unique property of HFNO—the capacity to remain in situ throughout laryngoscopy—retains particular value during the apneic interval regardless of which modality is chosen for pre-oxygenation.

Disease-specific physiology modifies this general framework in clinically meaningful ways. Patients with chronic CO_2_ retention require conservative apneic time limits and mandatory CO_2_ monitoring. Patients with decompensated heart failure or severe restrictive lung disease may benefit from awake tracheal intubation when induction-related cardiovascular compromise is anticipated. Patients with severe obesity, OSA, or obesity-hypoventilation syndrome require pressure-supported pre-oxygenation in the ramped position regardless of the specific modality selected.

The translation of this evidence into consistent practice remains incomplete, with persistent under-implementation of NIV-based pre-oxygenation in intensive care units worldwide and substantial inter-institutional variability in HFNO use. Closing this implementation gap—through education, equipment availability, and protocolization tailored to local resources—is a clinical priority that the available evidence is now mature enough to support. Ultimately, peri-intubation oxygenation exemplifies personalized medicine in acute care: optimal outcomes depend less on any single dominant technique than on individualizing modality, settings, and monitoring to each patient’s phenotype and physiological reserve.

## 11. Future Directions

Despite the rapid evolution of the peri-intubation oxygenation literature, several questions remain insufficiently answered to permit definitive recommendations across all clinical scenarios. Six research priorities emerge from the present synthesis.

Head-to-head trials of NIV versus HFNO versus combined NIV-plus-HFNO in well-defined AHRF phenotypes. The PREOXI and OPTINIV trials [9,11] have established the NIV-versus-facemask and combined-versus-NIV comparisons, respectively, but a direct three-arm comparison powered for clinically meaningful endpoints—peri-intubation desaturation, cardiac arrest, hemodynamic collapse, and 28-day mortality—would substantially clarify modality selection in severely hypoxemic patients. Such trials should pre-specify analysis by baseline PaO_2_/FiO_2_ stratum, since the relative benefit of each strategy almost certainly depends on baseline severity.

Disease-specific protocol optimization. Current recommendations for HFNO and NIV settings (flow rate, FiO_2_, pressure support) are largely extrapolated from mixed populations. Prospective studies should define optimal settings tailored to specific phenotypes—COPD, heart failure, ILD, obesity-hypoventilation syndrome, advanced ILD, and high-risk obstetrics—and should incorporate physiological endpoints (EELV, transpulmonary pressure, regional ventilation distribution) alongside clinical outcomes.

Long-term clinical outcomes beyond peri-intubation oxygenation. Most existing trials evaluate immediate oxygenation parameters or short-term peri-intubation events. Data on 28-day mortality, ventilator-free days, ICU and hospital length of stay, neurocognitive outcomes after traumatic brain injury, and long-term respiratory recovery are needed to fully characterize the clinical impact of peri-intubation oxygenation choices.

Integration of physiology-informed monitoring. As discussed, EIT, transcutaneous CO_2_, diaphragmatic ultrasound, and esophageal pressure monitoring represent investigational tools with strong physiological rationale but limited integration into routine peri-intubation practice. Trials incorporating these modalities—both as outcome measures and as titration tools—would advance the field toward truly personalized oxygenation strategies and align with the broader objective of physiology-informed critical care articulated in current guidelines and editorial calls.

Implementation science and quality improvement. The persistent under-use of NIV-based pre-oxygenation in ICUs worldwide [56] indicates that evidence alone does not translate into practice. Research addressing barriers to adoption, training requirements, and protocolization strategies—including simulation-based training and structured pre-intubation checklists—is essential to close the gap between evidence and practice.

Pre-hospital and emergency department settings. The bulk of high-quality peri-intubation oxygenation evidence derives from the ICU. The pre-hospital and emergency department settings, where intubation is frequent and resources are constrained, remain comparatively under-studied. The forthcoming results of Pre-AeRATE [29] will partially address this gap, but additional trials in pre-hospital and resource-limited settings are needed.

The peri-intubation phase exemplifies how non-pharmacological respiratory interventions, when grounded in physiological understanding and applied with attention to individual patient phenotype, can meaningfully improve outcomes in the most vulnerable patient populations. The next decade of research will likely shift the framing from “which modality is best?” to “which modality, at which settings, for which phenotype, monitored with which tools?”—a more refined question that the evolving evidence base is now equipped to address.

## Figures and Tables

**Table 1:** Summary of principal studies informing the present narrative synthesis, organized by phase of peri-intubation airway management.

Study (Ref.)	Year	Design	Population/Setting	Comparator	Key Outcome/Finding	OCEBM Level
Pre-Oxygenation						
Lodenius [13]	2018	RCT	Adults, RSI	Facemask	THRIVE non-inferior for safe apnea time	2
Sjöblom [14]	2026	RCT	Elective surgical adults	Variable HFNO flows	Optimal HFNO flow ≈ 45 L·min^−1^	2
Wu [15]	2022	RCT	Obese adults, elective intubation	Facemask	Higher PaO_2_, fewer desaturations with HFNO	2
Schutzer-Weissmann [16]	2023	RCT	Morbid obesity	Facemask	Safe apnea extended to 18 min with HFNO	2
Heinrich [17]	2014	RCT	Bariatric surgery	CPAP, facemask	HFNO improves PaO_2_ vs. facemask, non-inferior to CPAP	2
Pitre [10]	2025	Network MA	Critically ill, pre-oxygenation	Multiple	NIV ranked superior; HFNO intermediate; facemask inferior	1
Gibbs (PREOXI) [11]	2024	RCT (*n* = 1301)	Critically ill, emergency intubation	Oxygen mask	NIV halves incidence of severe desaturation (9.1% vs. 18.5%)	2
Jaber (OPTINIV) [9]	2016	RCT	Severely hypoxemic ICU	NIV alone	NIV + HFNO during apnea improves min SpO_2_ (100% vs. 96%)	2
Zhou [18]	2021	RCT	Pregnancy, RSII	Facemask	Superior maternal oxygenation with HFNO	2
Craig [19]	2025	SR	Obstetric, peri-operative	COT	HFNO benefit uncertain in obstetric setting	1
Apneic Oxygenation						
Patel (THRIVE) [20]	2015	OS	Difficult airway adults	Historical	Mean apnea 14 min without desaturation < 90%	4
Wong [21]	2019	RCT	BMI > 40 kg·m^−2^	COT	Safe apnea 4.3 vs. 1.3 min	2
Humphreys [22]	2017	RCT	Pediatric, normal airway	Facemask	THRIVE doubles safe apnea time	2
Riva [23]	2018	RCT	Pediatric apneic oxygenation	Conventional	Extended safe apnea time, faster CO_2_ rise	2
Jaber (OPTIMASK) [24]	2023	OS (international, *n* = 450)	General anesthesia, mixed BMI	Facemask alone	Higher EtO_2_ at intubation with facemask + HFNO	3
Awake Tracheal Intubation						
Badiger [25]	2015	OS	Awake fibreoptic intubation	None	HFNO improves oxygenation during ATI	4
Emergency/Trauma						
Russotto (INTUBE) [1]	2021	OS (international, 29 countries)	Critically ill, intubation	None	Peri-intubation cardiovascular collapse > 40%, hypoxemia 9%, cardiac arrest 3.1%	3
Raineri [26]	2017	OS	Urgent abdominal surgery, RSII	Pre/post baseline	Min SpO_2_ 96%, max apnea 12 min	4
Tang [27]	2025	SR/MA	Emergency RSI	Facemask	HFNO superior for oxygenation maintenance	1
White [28]	2023	SR (Cochrane)	Apneic oxygenation various settings	Multiple	Heterogeneous evidence; benefit signal in selected groups	1
Chua (Pre-AeRATE) [29]	2019	RCT protocol	Emergency RSI	Standard care	Ongoing trial, results expected	2
Mechanism/Physiology						
Möller [30]	2015	PS	Upper airway models	—	HFNO clears anatomical dead space	5
Parke [31]	2009	PS	Adult volunteers	—	HFNO generates low-level positive airway pressure	5
Riera [32]	2013	OS	Cohort with EIT	—	Increased end-expiratory lung volume with HFNO	4
Corley [33]	2011	OS	Post-cardiac surgery	—	Increased EELV, reduced respiratory rate	4
Natalini [34]	2019	PS	Tracheostomized patients	—	50 L·min^−1^ minimum for tracheal benefit	5
McGinley [35]	2007	PS	OSA volunteers	—	Nasal cannula at >35 L·min^−1^ prevents upper airway collapse	5

RCT: randomized controlled trial; SR/MA: systematic review and/or meta-analysis; OS: observational study; PS: physiological study; RSI/RSII: rapid sequence induction/intubation; EIT: electrical impedance tomography; EELV: end-expiratory lung volume. OCEBM levels of evidence (2011) are reported as a qualitative indication of study design, not as a formal quality grade.

**Table 2:** Technical and operational comparison of high-flow nasal oxygen (HFNO), conventional facemask oxygen therapy, and non-invasive ventilation (NIV) in the peri-intubation context.

Parameter	HFNO	Conventional Facemask	NIV (CPAP/BiPAP)
Flow rate (L·min^−1^)	20–70	6–15	Variable, demand-driven
FiO_2_	0.21–1.0	0.30–0.90	0.21–1.0
Humidification	Active (100% RH, 44 mg H_2_O·L^−1^)	Passive/absent	Variable
Gas temperature (°C)	34–37	Ambient	Ambient
Positive airway pressure (cmH_2_O)	2.7–7.4 (mouth closed)	0	5–15 PEEP + 5–15 PS
Interface	Wide-bore nasal cannulae	Facemask with reservoir	Full-facemask or helmet
Maintained in situ during laryngoscopy	Yes	No (must be removed)	No (must be removed)
Patient tolerability	+++	++	+
Effective in severe hypoxemia	Moderate	Limited	High

Patient tolerability is expressed on a semi-quantitative scale (+++ excellent, ++ good, + moderate). PEEP: positive end-expiratory pressure; PS: pressure support; RH: relative humidity.

**Table 3:** Formal guideline documents relevant to peri-intubation oxygenation in patients at risk of acute hypoxaemic respiratory failure. Entries summarise the scope and the stated positions of each document; no guideline currently specifies a PaO_2_/FiO_2_ threshold at which non-invasive ventilation should replace high-flow nasal oxygen.

Guideline (Year)	Population and Scope	Stated Position Relevant to Peri-Intubation Oxygenation	Ref.
ESA/ESICM joint guideline (2020)	Hypoxaemic adult patient in the perioperative and periprocedural period	Nineteen GRADE-based recommendations. The two graded 1B state that NIPPV or CPAP (according to local expertise) is preferred to COT for improving oxygenation and that NIPPV or CPAP should be used immediately post-extubation in hypoxaemic patients at risk of acute respiratory failure after abdominal surgery.	[43]
DAS/ICS/FICM/RCoA (2018)	Tracheal intubation in critically ill adults, in all hospital locations	Emphasises the primacy of oxygenation, including pre-oxygenation and peroxygenation, and recommends a modified rapid sequence approach. Stresses human factors, limitation of intubation attempts, early videolaryngoscopy, and prompt transition through the algorithm.	[58]
SIAARTI consensus (2016)	Perioperative and periprocedural airway management and respiratory safety in the obese patient	Consensus recommendations addressing airway management and respiratory safety specifically in the obese phenotype, including attention to positioning and pre-oxygenation.	[59]
OAA/DAS (2015)	Management of difficult and failed tracheal intubation in obstetrics	Guidance on obstetric airway management, in a population characterised by reduced functional residual capacity and accelerated desaturation.	[60]

CPAP, continuous positive airway pressure; COT, conventional oxygen therapy; NIPPV, non-invasive positive-pressure ventilation.

## Data Availability

No new data were created or analyzed in this study. Data sharing is not applicable to this article.

## Abbreviations

AHRF	acute hypoxemic respiratory failure
HFNO	high-flow nasal oxygen
NIV	non-invasive ventilation
CPAP	continuous positive airway pressure
BiPAP	bilevel positive airway pressure
FRC	functional residual capacity
FiO_2_	fraction of inspired oxygen
THRIVE	transnasal humidified rapid-insufflation ventilatory exchange
RSI	rapid sequence induction/intubation
ATI	awake tracheal intubation
COPD	chronic obstructive pulmonary disease
ILD	interstitial lung disease
OSA	obstructive sleep apnea
OHS	obesity-hypoventilation syndrome
EIT	electrical impedance tomography
TcCO_2_	transcutaneous carbon dioxide
BMI	body mass index

## References

[1] Russotto V., Myatra S.N., Laffey J.G., Tassistro E., Antolini L., Bauer P., Lascarrou J.B., Szułdrzyński K., Camporota L., Pelosi P. (2021). Intubation practices and adverse peri-intubation events in critically ill patients from 29 countries. JAMA.

[2] Russotto V., Tassistro E., Myatra S.N., Parotto M., Antolini L., Bauer P., Lascarrou J.B., Szułdrzyński K., Camporota L., Putensen C. (2022). Peri-intubation cardiovascular collapse in patients who are critically ill: Insights from the INTUBE Study. Am. J. Respir. Crit. Care Med..

[3] Russotto V., Laffey J.G., Tassistro E., Myatra S.N., Rezoagli E., Foti G., Antolini L., Valsecchi M.G., Bauer P.R., Szułdrzyński K. (2025). Peri-intubation complications in critically ill obese patients: A secondary analysis of the international INTUBE cohort. Crit. Care.

[4] Karamchandani K., Nasa P., Jarzebowski M., Brewster D.J., De Jong A., Bauer P.R., Berkow L., Brown C.A., Cabrini L., Casey J. (2024). Tracheal intubation in critically ill adults with a physiologically difficult airway: An international Delphi study. Intensive Care Med..

[5] Mosier J.M., Joshi R., Hypes C., Pacheco G., Valenzuela T., Sakles J.C. (2015). The physiologically difficult airway. West J. Emerg. Med..

[6] Cortegiani A., Accurso G., Mercadante S., Giarratano A., Gregoretti C. (2018). High flow nasal therapy in perioperative medicine: From operating room to general ward. BMC Anesthesiol..

[7] Renda T., Corrado A., Iskandar G., Pelaia G., Abdalla K., Navalesi P. (2018). High-flow nasal oxygen therapy in intensive care and anaesthesia. Br. J. Anaesth..

[8] Park S.Y. (2021). High-flow nasal cannula for respiratory failure in adult patients. Acute Crit. Care.

[9] Jaber S., Monnin M., Girard M., Conseil M., Cisse M., Carr J., Mahul M., Delay J.M., Belafia F., Chanques G. (2016). Apnoeic oxygenation via high-flow nasal cannula oxygen combined with non-invasive ventilation preoxygenation for intubation in hypoxaemic patients in the intensive care unit: The single-centre, blinded, randomised controlled OPTINIV trial. Intensive Care Med..

[10] Pitre T., Liu W., Zeraatkar D., Casey J.D., Dionne J.C., Gibbs K.W., Ginde A.A., Needham-Nethercott N., Rice T.W., Semler M.W. (2025). Preoxygenation strategies for intubation of patients who are critically ill: A systematic review and network meta-analysis of randomised trials. Lancet Respir. Med..

[11] Gibbs K.W., Semler M.W., Driver B.E., Seitz K.P., Stempek S.B., Taylor C., Resnick-Ault D., White H.D., Gandotra S., Doerschug K.C. (2024). Noninvasive ventilation for preoxygenation during emergency intubation. N. Engl. J. Med..

[12] OCEBM Levels of Evidence Working Group (2011). The Oxford 2011 Levels of Evidence.

[13] Lodenius Å., Piehl J., Östlund A., Ullman J., Jonsson Fagerlund M. (2018). Transnasal humidified rapid-insufflation ventilatory exchange (THRIVE) vs. facemask breathing pre-oxygenation for rapid sequence induction in adults: A prospective randomised non-blinded clinical trial. Anaesthesia.

[14] Sjöblom A., Hoffman F., Hedberg M., Forsberg I.-M., Fagerlund M.J. (2026). Preoxygenation with high-flow nasal oxygen at various flow rates in elective surgical patients: A prospective, randomised, single-blind clinical trial. Br. J. Anaesth..

[15] Wu Y.M., Li C.C., Huang S.Y., Su Y.-H., Wang C.-W., Chen J.-T., Shen S.-C., Lo P.-H., Yang Y.-L., Cherng Y.-G. (2022). A comparison of oxygenation efficacy between high-flow nasal cannulas and standard facemasks during elective tracheal intubation for patients with obesity: A randomized controlled trial. J. Clin. Med..

[16] Schutzer-Weissmann J., Wojcikiewicz T., Karmali A., Lukosiute A., Sun R., Kanji R., Ahmed A.R., Purkayastha S., Brett S.J., Cousins J. (2023). Apnoeic oxygenation in morbid obesity: A randomised controlled trial comparing facemask and high-flow nasal oxygen delivery. Br. J. Anaesth..

[17] Heinrich S., Horbach T., Stubner B., Prottengeier J., Irouschek A., Schmidt J. (2014). Benefits of heated and humidified high flow nasal oxygen for preoxygenation in morbidly obese patients undergoing bariatric surgery: A randomized controlled study. J. Obes. Bariatr..

[18] Zhou S., Zhou Y., Cao X., Ni X., Du W., Xu Z., Liu Z. (2021). The efficacy of high flow nasal oxygenation for maintaining maternal oxygenation during rapid sequence induction in pregnancy: A prospective randomised clinical trial. Eur. J. Anaesthesiol..

[19] Craig R., Curran L., Bampoe S., Odor P., Blake L., Carvalho B., O’Carroll J. (2025). Peri-operative use of high-flow nasal oxygen in obstetric patients: A systematic review. Anaesthesia.

[20] Patel A., Nouraei S.A.R. (2015). Transnasal humidified rapid-insufflation ventilatory exchange (THRIVE): A physiological method of increasing apnoea time in patients with difficult airways. Anaesthesia.

[21] Wong D.T., Dallaire A., Singh K.P., Madhusudan P., Jackson T., Singh M., Wong J., Chung F. (2019). High-flow nasal oxygen improves safe apnea time in morbidly obese patients undergoing general anesthesia: A randomized controlled trial. Anesth. Analg..

[22] Humphreys S., Lee-Archer P., Reyne G., Long D., Williams T., Schibler A. (2017). Transnasal humidified rapid-insufflation ventilatory exchange (THRIVE) in children: A randomized controlled trial. Br. J. Anaesth..

[23] Riva T., Pedersen T.H., Seiler S., Kasper N., Theiler L., Greif R., Kleine-Brueggeney M. (2018). Transnasal humidified rapid insufflation ventilatory exchange for oxygenation of children during apnoea: A prospective randomised controlled trial. Br. J. Anaesth..

[24] Jaber S., De Jong A., Schaefer M.S., Zhang J., Ma X., Hao X., Zhou S., Lv S., Banner-Goodspeed V., Niu X. (2023). Preoxygenation with standard facemask combining apnoeic oxygenation using high flow nasal cannula versus standard facemask alone in patients with and without obesity: The OPTIMASK international study. Ann. Intensive Care.

[25] Badiger S., John M., Fearnley R.A., Ahmad I. (2015). Optimizing oxygenation and intubation conditions during awake fibre-optic intubation using a high-flow nasal oxygen-delivery system. Br. J. Anaesth..

[26] Raineri S.M., Cortegiani A., Accurso G., Procaccianti C., Vitale F., Caruso S., Giarrjatano A., Gregoretti C. (2017). Efficacy and safety of using high-flow nasal oxygenation in patients undergoing rapid sequence intubation. Turk. J. Anaesthesiol. Reanim..

[27] Tang H., Yang Y., Li H. (2025). High-flow nasal cannula for pre- and apneic oxygenation during rapid sequence induction intubation in emergency surgery: A systematic review and meta-analysis. PLoS ONE.

[28] White L.D., Vlok R.A., Thang C.Y.C., Tian D.H., Melhuish T.M. (2023). Oxygenation during the apnoeic phase preceding intubation in adults in prehospital, emergency department, intensive care and operating theatre environments. Cochrane Database Syst. Rev..

[29] Chua M.T., Khan F.A., Ng W.M., Lu Q., Low M.J.W., Yau Y.W., Punyadasa A., Kuan W.S. (2019). Pre- and apnoeic high flow oxygenation for RApid sequence intubation in The Emergency department (Pre-AeRATE): Study protocol for a multicentre, randomised controlled trial. Trials.

[30] Möller W., Celik G., Feng S., Bartenstein P., Meyer G., Eickelberg O., Schmid O., Tatkov S. (2015). Nasal high flow clears anatomical dead space in upper airway models. J. Appl. Physiol..

[31] Parke R., McGuinness S., Eccleston M. (2009). Nasal high-flow therapy delivers low level positive airway pressure. Br. J. Anaesth..

[32] Riera J., Pérez P., Cortés J., Roca O., Masclans J.R., Rello J. (2013). Effect of high-flow nasal cannula and body position on end-expiratory lung volume: A cohort study using electrical impedance tomography. Respir. Care.

[33] Corley A., Caruana L.R., Barnett A.G., Tronstad O., Fraser J.F. (2011). Oxygen delivery through high-flow nasal cannulae increase end-expiratory lung volume and reduce respiratory rate in post-cardiac surgical patients. Br. J. Anaesth..

[34] Natalini D., Grieco D.L., Santantonio M.T., Mincione L., Toni F., Anzellotti G.M., Eleuteri D., Di Giannatale P., Antonelli M., Maggiore S.M. (2019). Physiological effects of high-flow oxygen in tracheostomized patients. Ann. Intensive Care.

[35] McGinley B.M., Patil S.P., Kirkness J.P., Smith P.L., Schwartz A.R., Schneider H. (2007). A nasal cannula can be used to treat obstructive sleep apnea. Am. J. Respir. Crit. Care Med..

[36] Sorbello M., Paternò D.S., Zdravkovic I., La Via L. (2025). Pharmacological approach to rapid sequence induction/intubation: A contemporary perspective. Curr. Opin. Anaesthesiol..

[37] Nishimura M. (2015). High-flow nasal cannula oxygen therapy in adults. J. Intensive Care.

[38] Nishimura M. (2016). High-flow nasal cannula oxygen therapy in adults: Physiological benefits, indication, clinical benefits, and adverse effects. Respir. Care.

[39] Groves N., Tobin A. (2007). High flow nasal oxygen generates positive airway pressure in adult volunteers. Aust. Crit. Care.

[40] Chanques G., Constantin J.M., Sauter M., Jung B., Sebbane M., Verzilli D., Lefrant J.-Y., Jaber S. (2009). Discomfort associated with underhumidified high-flow oxygen therapy in critically ill patients. Intensive Care Med..

[41] Salah B., Dinh Xuan A., Fouilladieu J., Lockhart A., Regnard J. (1988). Nasal mucociliary transport in healthy subjects is slower when breathing dry air. Eur. Respir. J..

[42] Jaber S., Chanques G., Jung B. (2010). Postoperative noninvasive ventilation. Anesthesiology.

[43] Leone M., Einav S., Chiumello D., Constantin J.-M., De Robertis E., De Abreu M.G., Gregoretti C., Jaber S., Maggiore S.M., Pelosi P. (2020). Noninvasive respiratory support in the hypoxaemic peri-operative/periprocedural patient: A joint ESA/ESICM guideline. Eur. J. Anaesthesiol..

[44] La Via L., Cuttone G., Senussi Testa T., Duarte-Medrano G., Nuno-Lambarri N., Deana C., Maniaci A., Paternò D.S., Zdravkovic I., Sorbello M. (2025). Non-invasive positive pressure ventilation for pre-oxygenation of critically ill patients before intubation. J. Clin. Med..

[45] Choi H., Lee J., Song J. (2016). Steam burn on nose by heated, humidified high-flow nasal cannula in neonate. Int. Wound J..

[46] Jasin L.R., Kern S., Thompson S., Walter C., Rone J.M., Yohannan M.D. (2008). Subcutaneous scalp emphysema, pneumo-orbitis and pneumocephalus in a neonate on high humidity high flow nasal cannula. J. Perinatol..

[47] Iglesias-Deus A., Perez-Munuzuri A., Lopez-Suarez O., Crespo P., Couce M.L. (2017). Tension pneumocephalus induced by high-flow nasal cannula ventilation in a neonate. Arch. Dis. Child. Fetal Neonatal Ed..

[48] Tanoubi I., Drolet P., Donati F. (2009). Optimizing preoxygenation in adults. Can. J. Anaesth..

[49] Pourmand A., Robinson C., Dorwart K., O’Connell F. (2017). Pre-oxygenation: Implications in emergency airway management. Am. J. Emerg. Med..

[50] Hodgson L.E., Murphy P.B., Hart N. (2015). Respiratory management of the obese patient undergoing surgery. J. Thorac. Dis..

[51] Mort T.C. (2005). Preoxygenation in critically ill patients requiring emergency tracheal intubation. Crit. Care Med..

[52] Grude O., Solli H.J., Andersen C., Hoff I.H., Klepstad P. (2018). Effect of nasal or nasopharyngeal apneic oxygenation on desaturation during induction of anesthesia and endotracheal intubation in the operating room: A narrative review of randomized controlled trials. J. Clin. Anesth..

[53] Vourc’h M., Huard D., Le Penndu M., Deransy R., Surbled M., Malidin M., Mahe P.-J., Guitton C., Roquilly A., Malard O. (2023). High-flow oxygen therapy versus facemask preoxygenation in anticipated difficult airway management (PREOPTI-DAM): An open-label, single-centre, randomised controlled phase 3 trial. eClinicalMedicine.

[54] Figg W., Butler H., Gibbs K.W., Casey J.D., Semler M.W., Brainard J., Douin D., Marvi T., Resnick-Ault D., Ginde A. (2026). Effect modification by pulmonary comorbidity: A secondary analysis of the PREOXI trial. Am. J. Respir. Crit. Care Med..

[55] Baillard C., Fosse J.P., Sebbane M., Chanques G., Vincent F., Courouble P., Cohen Y., Eledjam J.-J., Adnet F., Jaber S. (2006). Noninvasive ventilation improves preoxygenation before intubation of hypoxic patients. Am. J. Respir. Crit. Care Med..

[56] Crimi C., Noto A., Cortegiani A., Carlucci A., Gregoretti C., Inal-Ince D., Franssen F.M.E.M.E., Karagiannidis C., Winck J.C., Fisser C. (2025). Practices of high-flow nasal therapy in acute and chronic respiratory failure: The Hi-Flow Survey. BMJ Open Respir. Res..

[57] Frat J.P., Ricard J.D., Quenot J.P., Pichon N., Demoule A., Forel J.-M., Mira J.-P., Coudroy R., Berquier G., Voisin B. (2019). Non-invasive ventilation versus high-flow nasal cannula oxygen therapy with apnoeic oxygenation for preoxygenation before intubation of patients with acute hypoxaemic respiratory failure: A randomised, multicentre, open-label trial. Lancet Respir. Med..

[58] Higgs A., McGrath B.A., Goddard C., Rangasami J., Suntharalingam G., Gale R., Cook T. (2018). Guidelines for the management of tracheal intubation in critically ill adults. Br. J. Anaesth..

[59] Petrini F., Di Giacinto I., Cataldo R., Esposito C., Pavoni V., Donato P., Trolio A., Merli G., Sorbello M., Pelosi P. (2016). Perioperative and periprocedural airway management and respiratory safety for the obese patient: 2016 SIAARTI Consensus. Minerva Anestiol..

[60] Mushambi M.C., Kinsella S.M., Popat M., Swales H., Ramaswamy K.K., Winton A.L., Quinn A.C. (2015). Obstetric Anaesthetists’ Association and Difficult Airway Society guidelines for the management of difficult and failed tracheal intubation in obstetrics. Anaesthesia.

[61] Steier J., Jolley C.J., Seymour J., Roughton M., Polkey M.I., Moxham J. (2009). Neural respiratory drive in obesity. Thorax.

[62] Tan P.C.F., Dennis A.T. (2018). High flow humidified nasal oxygen in pregnant women. Anaesth. Intensive Care.

[63] Fayed M., Maroun W., Patel N., Galusca D. (2023). Apneic oxygenation: A summarized review and stepwise approach. Cureus.

[64] Lyons C., Callaghan M. (2019). Uses and mechanisms of apnoeic oxygenation: A narrative review. Anaesthesia.

[65] Hardman J.G., Wills J.S. (2006). The development of hypoxaemia during apnoea in children: A computational modelling investigation. Br. J. Anaesth..

[66] Riva T., Seiler S., Stucki F., Greif R., Theiler L. (2016). High-flow nasal cannula therapy and apnea time in laryngeal surgery. Paediatr. Anaesth..

[67] Stock M.C., Schisler J.Q., McSweeney T.D. (1989). The PaCO_2_ rate of rise in anesthetized patients with airway obstruction. J. Clin. Anesth..

[68] Gustafsson I.M., Lodenius Å., Tunelli J., Ullman J., Jonsson Fagerlund M. (2017). Apnoeic oxygenation in adults under general anaesthesia using Transnasal Humidified Rapid-Insufflation Ventilatory Exchange (THRIVE): A physiological study. Br. J. Anaesth..

[69] Cheng Q., Zhang J., Wang H., Zhang R., Yue Y., Li L. (2015). Effect of acute hypercapnia on outcomes and predictive risk factors for complications among patients receiving bronchoscopic interventions under general anesthesia. PLoS ONE.

[70] Laviola M., Das A., Chikhani M., Bates D.G., Hardman J.G. (2019). Computer simulation clarifies mechanisms of carbon dioxide clearance during apnoea. Br. J. Anaesth..

[71] Thiruvenkatarajan V., Jeyadoss J., Harford P., Kim M., Jayaraj A.K. (2026). High-flow nasal oxygen and the risk of gastric insufflation: A systematic review and meta-analysis supplemented by narrative synthesis. Anesth. Analg..

[72] La Via L., Iannella G., Pace A., Magliulo G., Cuttone G., Modica R., Lentini M., Botto C.G., Paternò D.S., Sorbello M. (2025). Anesthesiologic management of adult and pediatric patients with obstructive sleep apnea. Healthcare.

[73] Kotwinski D., Paton L., Langford R. (2018). The role of high flow nasal oxygen therapy in anaesthesia. Br. J. Hosp. Med..

[74] Russotto V., Cortegiani A., Raineri S.M., Gregoretti C., Giarratano A. (2017). Respiratory support techniques to avoid desaturation in critically ill patients requiring endotracheal intubation: A systematic review and meta-analysis. J. Crit. Care.

[75] Binks M.J., Holyoak R.S., Melhuish T.M., Vlok R., Bond E., White L.D. (2017). Apneic oxygenation during intubation in the emergency department and during retrieval: A systematic review and meta-analysis. Am. J. Emerg. Med..

[76] Lentz S., Grossman A., Koyfman A., Long B. (2020). High-risk airway management in the emergency department. Part I: Diseases and approaches. J. Emerg. Med..

[77] Ebeling C.G., Riccio C.A. (2019). Apneic oxygenation with high-flow nasal cannula and transcutaneous carbon dioxide monitoring during airway surgery: A case series. A A Pract..

